# Neonatal Restriction of Tactile Inputs Leads to Long-Lasting Impairments of Cross-Modal Processing

**DOI:** 10.1371/journal.pbio.1002304

**Published:** 2015-11-24

**Authors:** Kay Sieben, Malte Bieler, Brigitte Röder, Ileana L. Hanganu-Opatz

**Affiliations:** 1 Developmental Neurophysiology, Institute of Neuroanatomy, University Medical Center Hamburg-Eppendorf, Hamburg, Germany; 2 Biological Psychology and Neuropsychology, University Hamburg, Hamburg, Germany; Glasgow University, UNITED KINGDOM

## Abstract

Optimal behavior relies on the combination of inputs from multiple senses through complex interactions within neocortical networks. The ontogeny of this multisensory interplay is still unknown. Here, we identify critical factors that control the development of visual-tactile processing by combining in vivo electrophysiology with anatomical/functional assessment of cortico-cortical communication and behavioral investigation of pigmented rats. We demonstrate that the transient reduction of unimodal (tactile) inputs during a short period of neonatal development prior to the first cross-modal experience affects feed-forward subcortico-cortical interactions by attenuating the cross-modal enhancement of evoked responses in the adult primary somatosensory cortex. Moreover, the neonatal manipulation alters cortico-cortical interactions by decreasing the cross-modal synchrony and directionality in line with the sparsification of direct projections between primary somatosensory and visual cortices. At the behavioral level, these functional and structural deficits resulted in lower cross-modal matching abilities. Thus, neonatal unimodal experience during defined developmental stages is necessary for setting up the neuronal networks of multisensory processing.

## Abstract 2

Reducing unisensory experience during neonatal development causes permanent disruption of connectivity between primary sensory cortices, resulting in impaired multisensory abilities.

## Author Summary

Our senses, working together, enable us to interact with the environment. To obtain a unified percept of the world, diverse sensory inputs need to be bound together within distributed but strongly interconnected neuronal networks. Many multisensory abilities emerge or mature late in life, long after the maturation of the individual senses, yet the factors and mechanisms controlling their development are largely unknown. Here, we provide evidence for the critical role of unisensory experience during early postnatal life for the development of multisensory integration. Focusing on visual-tactile interactions in pigmented rats with good visual acuity, we show that a transient reduction of tactile inputs during neonatal development leads to sparser direct connections between adult primary visual and somatosensory cortices. As a result, these animals showed reduced neuronal activation following co-occurring tactile and visual stimuli, as well as impaired communication within visual-somatosensory networks. The structural and functional deficits resulting from an early manipulation of tactile experience had major behavioral consequences, impairing the rats’ ability to transfer information about encountered objects between senses. Thus, unisensory experience during early development shapes the neuronal networks of multisensory processing and the ability to transfer cross-modal information.

## Introduction

Most environmental events provide inputs to multiple senses that need to be integrated into a unified percept. Multisensory interactions have been observed both in higher-order brain regions and in primary sensory cortices [1–4]. Exposure to cross-modal stimuli has been shown to modulate the neuronal firing of cortical neurons and to shape the power and phase of oscillatory network activity [5–7]. Direct cortico-cortical connectivity [8] and feed-forward projections from thalamic nuclei [9] represent possible anatomical substrates of efficient multisensory processing at the cortical level. Furthermore, this multisensory processing in primary sensory areas supports the decoding of behaviorally relevant information and thereby improves task performance [10].

While the multisensory abilities are indispensable for daily life, their emergence is a progressive and protracted process that continues well after the development of individual senses [11,12]. For example, one of the earliest forms of multisensory integration in humans has been described at two months of age, when vowel information in faces and voices are matched [13]. In kittens, the first multisensory neurons were detected in the superior colliculus (SC) during the second postnatal week [14]. However, other multisensory processes do not reach adult level until adolescence [15,16]. The crucial requirements and processes controlling the development of multisensory interactions at the level of primary sensory cortices remain mechanistically unsolved. This knowledge gap is particularly striking in rodents, which have been shown to express highly multisensory abilities at the level of primary sensory cortices [8].

In contrast to multisensory maturation, the mechanisms underlying the unisensory development are better understood. The processing of unimodal stimuli is considered to rely on a highly precise wiring of the corresponding neuronal networks in the primary sensory cortices. The maturation of the neural circuitry is initiated under the control of molecular cues and subsequently refined by both experience-independent and experience-dependent electrical activity [17–20]. The experience-dependent refinement peaks during defined developmental stages, which have been termed critical or sensitive periods [21,22]. Unimodal inputs during these periods are crucial for shaping neuronal networks according to the environment and individual features. Hence, deprivation or reduction of unimodal input during this time results in abnormal patterns of neuronal activity in the corresponding primary sensory cortex and permanent behavioral impairment, whereas manipulation of the input before or after these periods is overall less detrimental [23,24]. While the development of multisensory processes in the SC neurons seems to depend on genuine cross-modal experience [25], it remains unclear which type of inputs controls the development of multisensory processing within neocortical networks.

Here, we aimed at elucidating the role of early unisensory experience for the development of multisensory interactions in the primary visual (V1) and somatosensory (S1) cortices as well as of multisensory object recognition in rats. We provide electrophysiological, anatomical, and behavioral evidence that neonatal unimodal inputs prior to the cross-modal experience are necessary for the correct maturation of multisensory processing by contributing to the setting up of structural and functional coupling within cortical networks.

## Results

**Fig 1:**
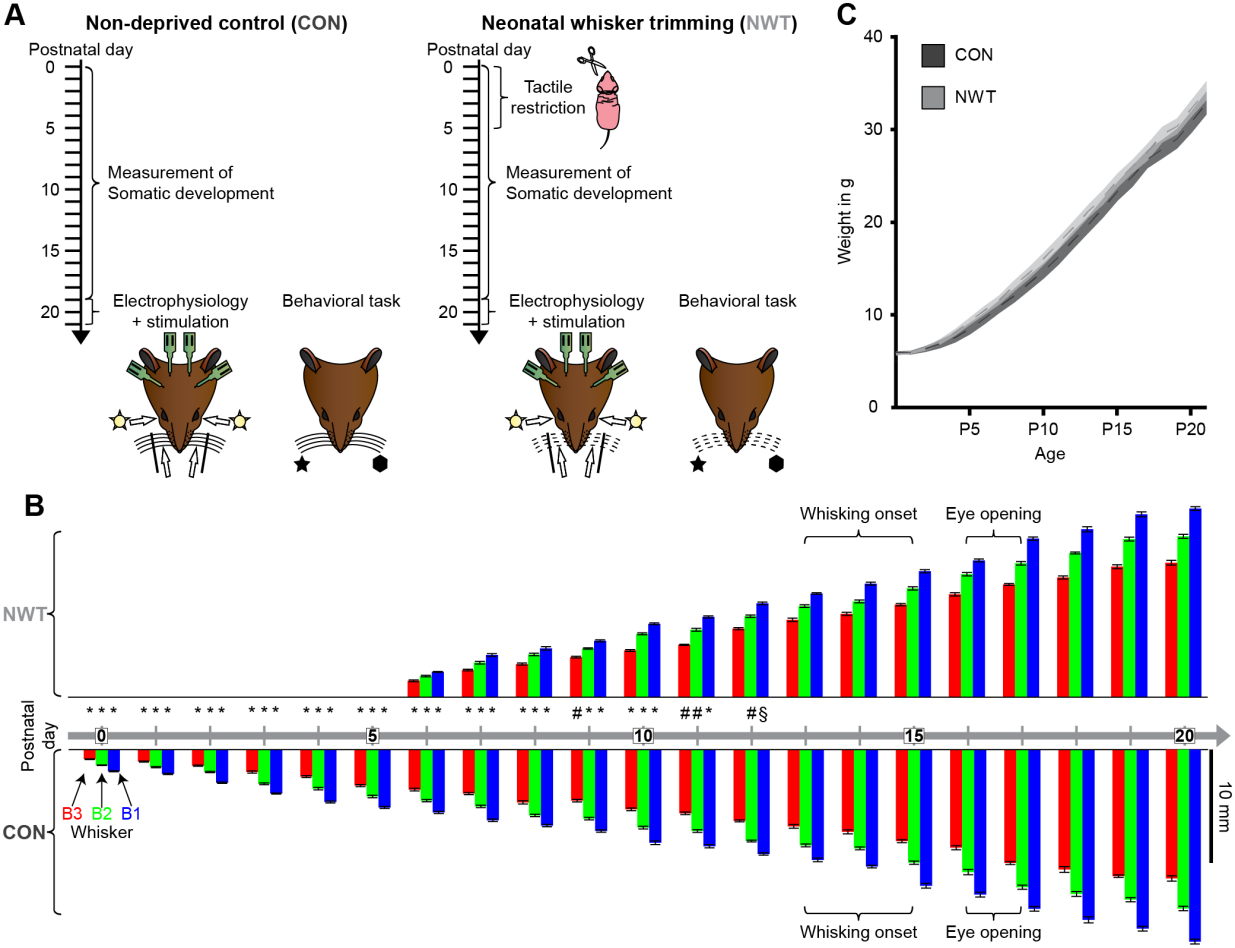
Experimental paradigm, whisker growth, and somatic development of CON and NWT rats. (**A**) Schematic drawing displaying the experimental protocol for the investigation of CON (left) and NWT (right) rats. (**B**) Mean length of three tracked whiskers (B1, B2, B3) averaged for CON (*n* = 10, bottom) and NWT (*n* = 8, top) rats and displayed for every day during the first three postnatal weeks. The onset of whisking behavior and eye opening are specified on the time line. Significant differences in the whisker length between CON and NWT rats are marked by § (*p* < 0.05), # (*p* < 0.01), and *(*p* < 0.001). (**C**) Plot displaying the weight increase of CON and NWT rats during the first three postnatal weeks. Dotted lines correspond to the mean and transparent area marks the SEM. The underlying data of this figure can be found in S1 Data. http://dx.doi.org/10.6084/m9.figshare.1540797

To assess the influence of unisensory experience during early development on the adult multisensory processing, the tactile input was transiently restricted (i.e., tactile receptors in the whisker pad are seldom and less precisely activated) by daily trimming the whiskers of pigmented Brown Norway rats from postnatal day (P) 0 to 5. This developmental time window has been shown to be critical for the maturation of the somatosensory system, because it corresponds to the developmental period of maximal ingrowth of thalamic axons into the cortical layer IV at topographically confined locations (barrels) [26]. In the following, the effects of neonatal tactile restriction on the multisensory processing at the neural and behavioral level were investigated in neonatally whisker-trimmed rats (NWT group, *n* = 27) and compared to age-matched (P19–22) non-deprived controls (CON group, *n* = 25) (Fig 1A).

To ensure that solely the early unisensory but not visual-tactile experience was manipulated, the trimmed vibrissae were allowed to regrow from P6 on. Already before the onset of light sensitivity in the retina (P12–13) and eye opening (P16–17) the whisker length did not significantly differ between NWT and CON rats (Fig 1B, S1A Table). Similarly, the adult-like whisking (amplitude >45°, frequency >5 Hz, duration >2 s) had a comparable (*p* = 0.37) onset in both groups of rats (14.5 ± 0.15 d after birth in CON rats versus 14.24 ± 0.18 d after birth in NWT rats). Moreover, their somatic development was similar (Fig 1C).

### Transient Neonatal Tactile Restriction Slightly Affects the Somatosensory Topography and Tactile Processing in S1

**Fig 2:**
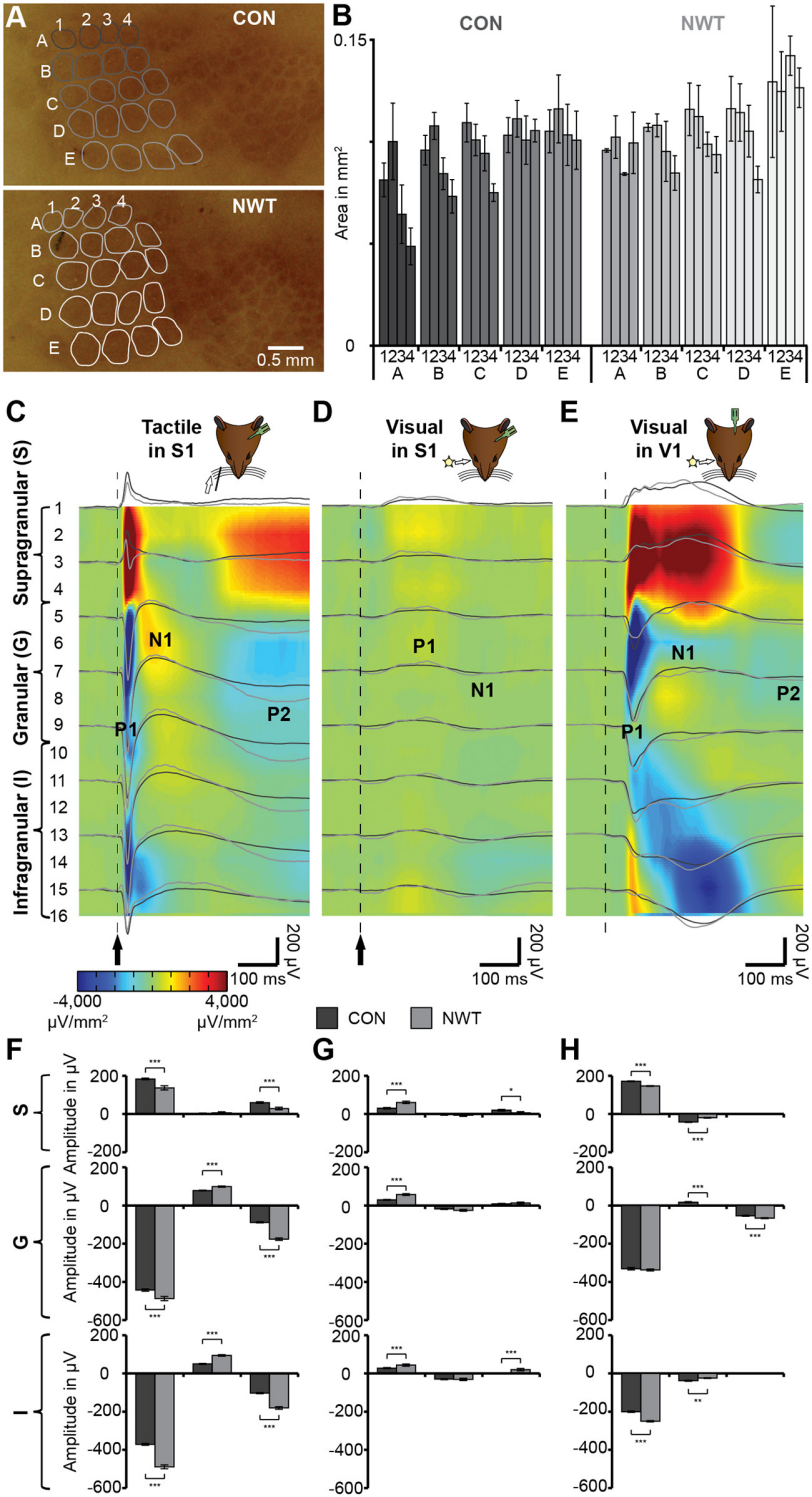
Effects of neonatal whisker trimming on the topographic organization of the adult barrel field and on unimodal evoked responses in S1 and V1. (**A**) Flattened histological sections including the posteromedial barrel field of the S1 stained for COX from a CON (top) and a NWT (bottom) rat. Barrel rows and columns are marked with letters and numbers. (**B**) Bar diagram displaying the size of individual barrels averaged for seven CON and seven NWT rats. (**C**) Averaged tactile potentials evoked by contralateral whisker deflection in S1 of CON (black traces) and NWT (gray traces) rats displayed together with the corresponding color-coded current source density (CSD) depth profile from CON rats. Numbers to the left mark the recording sites of the multielectrode array positioned in supragranular, granular and infragranular layers. For the evoked potential (EP) recorded in one channel in the granular layer the first three peaks (P1, N1, P2) are marked. (**D**) Averaged visual potentials evoked by contralateral light flashes in S1 of CON (black traces) and NWT (gray traces) rats displayed together with the corresponding color-coded CSD depth profile from CON rats. (**E**) Averaged visual potentials in V1 of CON (black traces) and NWT (gray traces) rats displayed together with the corresponding color-coded CSD depth profile from CON rats. (**F**) Bar diagrams displaying the mean amplitude of P1 (left), N1 (middle), and P2 (right) after tactile stimulation in supragranular (top), granular (middle) and infragranular (bottom) layers of the S1 in CON and NWT rats. (**G**) Same as (F) for visual stimulation and recordings in S1. (**H**) Same as (G) for visual stimulation and recordings in V1. The underlying data of this figure can be found in S1 Data. http://dx.doi.org/10.6084/m9.figshare.1540797

Since previous studies showed that unimodal experience during early development is crucial for the refinement of corresponding cortical networks and topographic maps, we assessed the impact of transient whisker trimming on the anatomy and tactile processing in S1. We firstly analyzed the structural changes in the barrel field of NWT rats post-mortem (Fig 2A). Similar to previous findings [27], the overall cytoarchitecture (i.e., number and position of barrels) of the posteromedial barrel subfield corresponding to the major whiskers was not affected by deprivation. The size of individual cytochrome oxidase-stained (COX) barrels was slightly increased in the NWT rats (*n* = 7) when compared to the corresponding barrels in CON rats (*n* = 7), but none of the differences reached significance level (Fig 2B, S1B Table).

Second, we characterized the effects of neonatal tactile restriction on unisensory evoked potentials (EPs) elicited by a mechanical deflection of the principal whiskers. Extracellular local field potentials (LFPs) were recorded simultaneously in S1 and V1 of lightly urethane-anesthetized NWT (*n* = 9) and CON (*n* = 10) rats using electrodes with 16 recording sites that covered the entire cortical depth. All recordings were conducted under urethane anesthesia to avoid the impact of varying alert states on multisensory processing [28]. Whisker deflection elicited in all cortical layers [supragranular (S), granular (G) and infragranular (I)] of the contralateral S1 of all investigated rats EPs with a depth profile characterized by layer specific polarity (Fig 2C). EPs consisted of a first prominent peak with positive surface polarity (P1) followed by additional negative and positive peaks (N1, P2). The absolute amplitude of tactile EPs in G and I layers was larger in NWT rats when compared to CON rats (Fig 2C and 2F). In contrast, the absolute amplitude of tactile EPs in the S layer was decreased in NWT compared to CON animals. Thus, the neonatal restriction caused specific changes of unimodal processing in the S1.

In line with our previous findings [8], the S1 responded to visual stimulation via light flashes, yet the evoked responses were significantly smaller when compared to tactile EPs and had the same polarity over all cortical layers, suggesting that they are at least partially volume conducted (e.g., non-specifically electrically spread at a distance from their source generator). However, these low-amplitude visually evoked responses in the S1 differed between CON and NWT rats (Fig 2D and 2G). Additionally, such plasticity was also induced for a non-deprived modality, since the EPs evoked by light flashes in the contralateral V1 differed between CON and NWT rats (Fig 2E and 2H).

### Cross-Modal Enhancement of Evoked Activity Critically Depends on Unimodal Inputs at Neonatal Age

The S1 seems to integrate simultaneously presented spatially congruent (i.e., in the same hemifield) whisker deflections and light flashes as reflected by the supra-additive enhancement of the EPs. Their magnitude was larger than the arithmetic sum of responses evoked by unimodal tactile and visual stimulations [8]. To assess the effects of neonatal tactile restriction on these multisensory interactions, we compared the bimodal EPs in NWT and CON rats.

**Fig 3:**
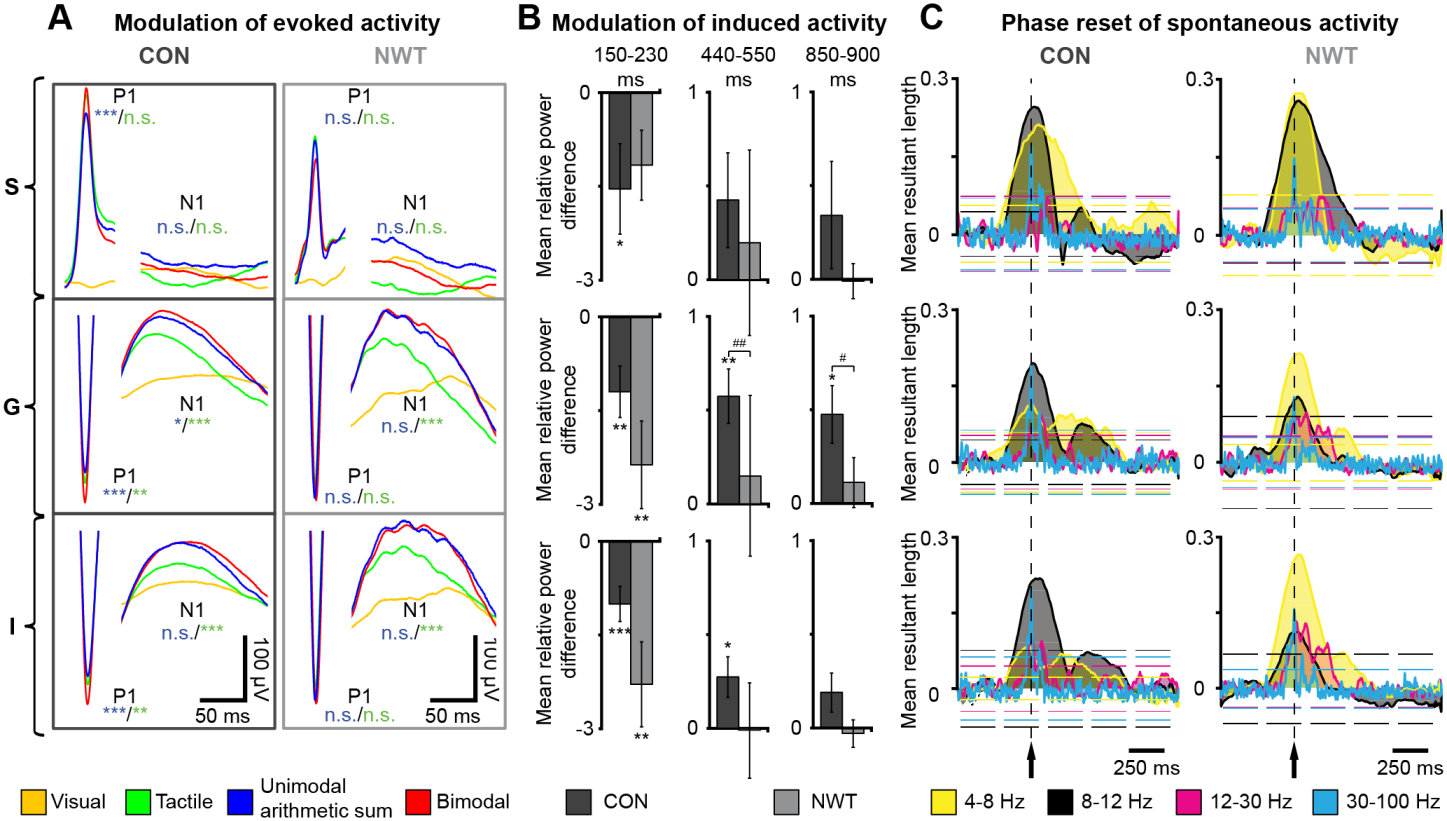
Cross-modal modulation of evoked and induced S1 network activity and visual phase reset of ongoing oscillatory activity in the S1 of CON and NWT rats. (**A**) Averaged potentials evoked by tactile (green), visual (orange), and bimodal (red) stimulation as well as the arithmetic sum of tactile and visual responses (blue) in the supragranular (top), granular (middle), and infragranular layers (bottom) of S1 in CON (left) and NWT rats (right). The first peak with positive surface polarity (P1) and the first negative peak (N1) are displayed. Significance values (not significant [n.s.], *p* < 0.05 [*], *p* < 0.01 [**], *p* < 0.001 [***]) correspond to the comparison of peak amplitudes between stimulation conditions (arithmetic sum of unimodal evoked responses versus congruent bimodal responses [blue]/tactile responses versus congruent bimodal responses [green]). (**B**) Bar diagrams displaying the mean relative power difference in gamma frequency range between bimodal and unimodal stimulation when calculated during different time windows post-stimulus in the S (top), G (middle), and I (bottom) layers of S1 in CON (black) and NWT rats (gray). Significance values correspond to the comparison between uni- and bimodal stimulation conditions (*) as well as between CON and NWT rats (#). (**C**) Plots displaying the mean resultant vector length of oscillatory phases in S1 of CON (left) and NWT rats (right) after contralateral visual stimulation (dotted line, black arrow). The values were averaged over time and layers in all CON and NWT rats for four distinct frequency bands: theta (4–8 Hz) in yellow, alpha (8–12 Hz) in black, beta (12–30 Hz) in magenta and gamma (30–100 Hz) in cyan. The dashed lines mark the borders of confidence interval for α = 0.01. The underlying data of this figure can be found in S1 Data. http://dx.doi.org/10.6084/m9.figshare.1540797

In CON rats, a supra-additive enhancement of the P1 peak after congruent visual-tactile stimulation was detected in all cortical layers when compared to unimodal stimulation (Fig 3A, S1C Table). Most prominently, in the G layer the absolute amplitude of multisensory P1 peak (581.8 ± 6.9 μV) was larger when compared to the unisensory response (552.9 ± 7.1 μV, *p* = 0.003) and the arithmetic sum of EPs resulting from tactile and visual stimulation (536.9 ± 8.0 μV, *p* < 0.001). Here, also the amplitude of the N1 peak was significantly augmented by bimodal stimulation (133.1 ± 2.5 μV versus unimodal: 98.6 ± 2.7 μV, *p* = 0.001 and arithmetic sum: 123.5 ± 3.8 μV, *p* = 0.016), exceeding the enhancing effect of unimodal visual stimulation on the tactile response. In contrast, this multisensory EP enhancement was significantly reduced in NWT rats. The absolute P1 amplitude in the G layer was similar after uni- and cross-modal stimulation (tactile, 621.6 ± 13.5 μV; unimodal arithmetic sum, 641.0 ± 14.8 μV; bimodal, 644.9 ± 13.4 μV; *p* = 0.73, *p* = 0.95). Moreover, the multisensory enhancement of the N1 peak (167.2 ± 4.4 μV) reached significance level only when compared to tactile stimulation (124.4 ± 4.4 μV, *p* < 0.001), but not when compared to the arithmetic sum of unimodal stimulations (166.0 ± 7.9 μV, *p* = 0.38). However, we cannot fully exclude the possibility that this small multisensory effect in NWT rats partially resulted from the volume-conducted visual responses in S1 (s. above). In any case, it can be concluded that neonatal whisker trimming diminished the multisensory enhancement of evoked activity in S1.

### Modulation of Cross-Modal Induced Activity and Visual Phase Reset of Spontaneous S1 Activity Critically Depends on Unimodal Inputs at Neonatal Age

Besides affecting the evoked activity, cross-modal stimulation has been reported to influence the cortical networks by modulating the stimulus-induced oscillatory activity (i.e., related but not locked to the stimulus) and the spontaneous ongoing oscillations (i.e., not causally related to the stimulus) [8,29]. To get first insights into the ontogeny of these modulatory processes, we quantified the multisensory power change of induced oscillatory activity at different time points after the stimulus onset for both NWT (*n* = 9) and CON rats (*n* = 10). Baseline-normalized wavelet spectra were used to calculate the power of oscillatory activity. For time windows of significant power change between tactile and bimodal stimulation (150–230 ms, 440–550 ms, and 850–900 ms post-stimulus) the differences between the two conditions were calculated and averaged (S1 Fig). As previously reported [8], the strongest modulatory effect of congruent bimodal stimuli on the power of induced activity of CON rats has been observed for the G layer (150–230 ms: 1.20 ± 0.41 times higher unisensory versus multisensory, *p* = 0.002; 440–550 ms: 0.57 ± 0.15 times higher multisensory versus unisensory, *p* = 0.002; 850–900 ms: 0.47 ± 0.15 times higher multisensory versus unisensory, *p* = 0.015). Power modulation after congruent bimodal stimulation was equally detected in NWT rats (150–230 ms: 2.36 ± 0.70 times higher unisensory versus multisensory, *p* = 0.002; 440–550 ms: 0.15 ± 0.43 times higher multisensory versus unisensory, *p* = 0.6; 850–900 ms: 0.11 ± 0.13 times higher multisensory versus unisensory, *p* = 0.43) (S1C Table). However, its magnitude, especially at later time windows post-stimulus, was lower. These changes were most prominent in the G layer (440–550 ms: *p* = 0.006; 850–900 ms: *p* = 0.025) (Fig 3B). The modulation of the power of oscillatory activity in CON and NWT rats was not accompanied by changes in population firing of S1 neurons. Analysis of the multi-unit activity (MUA) pre- and post-stimulus revealed the absence of multisensory effects in both groups of rats (*n* = 10 CON rats, *n* = 9 NWT rats) (S2 Fig).

Next, we assessed the effects of neonatal tactile restriction on the ongoing spontaneous activity (i.e., network activity non-related to the stimulus) in the S1. Cross-modal phase reset of network oscillations has been identified as a major cortical mechanism of multisensory interactions [5,6]. It has been hypothesized that timing of the neuronal activity by such a phase reset increases the processing efficiency of the stimulus [30]. In CON rats (*n* = 9), contralateral visual stimuli induced a prominent phase reset in all S1 layers that was detected as a concentration of a specific oscillatory phase in the histograms of phase resultant vector length (S3 Fig). Among the different frequency bands of oscillatory activity, the strongest phase reset was confined to 8–12 Hz (Fig 3C). For NWT rats (*n* = 10), the phase reset persisted, yet switched to a lower frequency (4–8 Hz) (Fig 3C).

Thus, neonatal tactile restriction altered multisensory processing in the adult S1 by perturbing the power modulation and the phase reset of network oscillations.

### Emergence of Cortico-cortical Connectivity and Functional Communication between Primary Sensory Cortices Requires Unimodal Inputs at Neonatal Age

**Fig 4:**
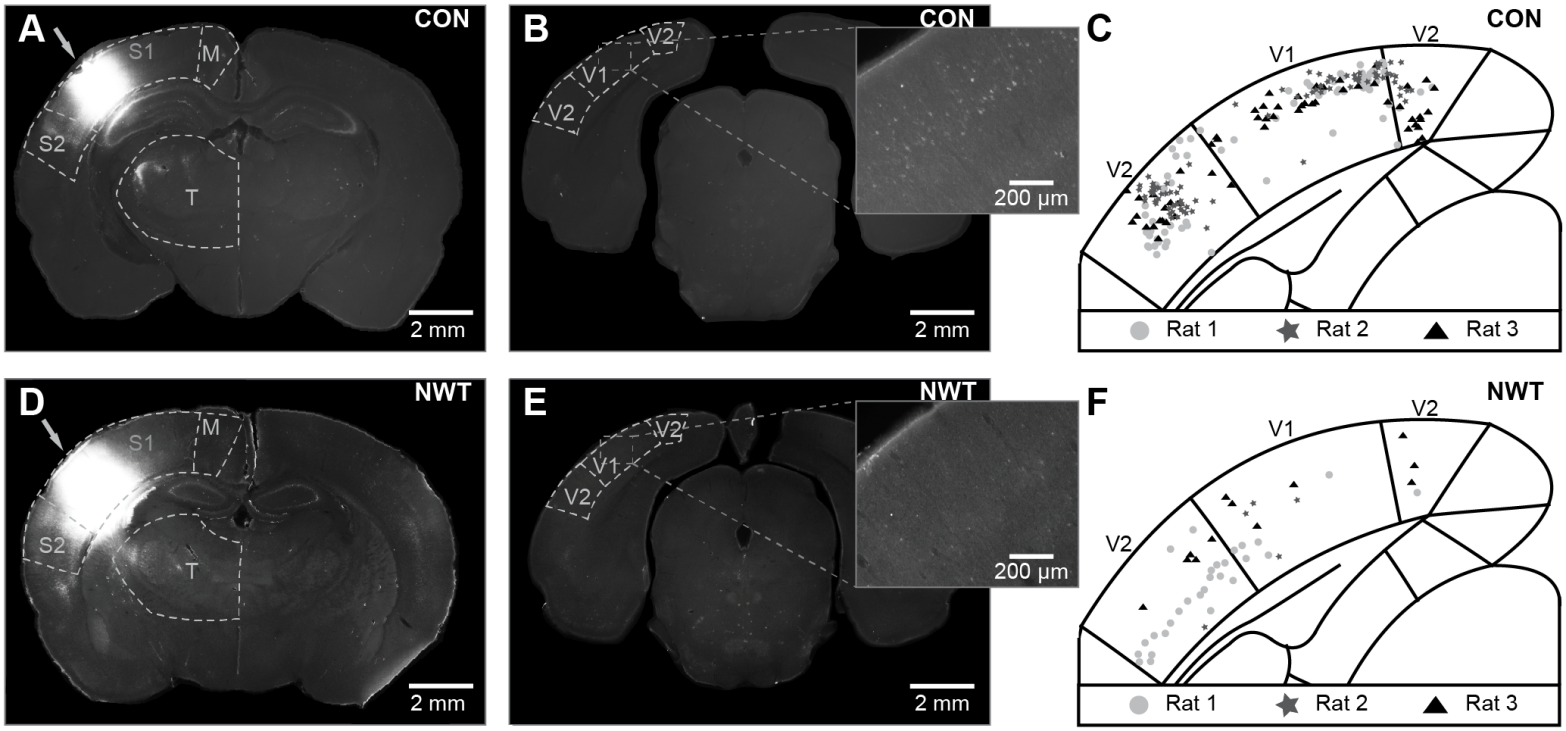
Direct connectivity between S1 and V1 of CON and NWT rats revealed by retrograde tracing with fluorogold. (**A**) Photomicrograph depicting FG spreading from the injection site (arrow) over all S1 layers in a 100 μm-thick coronal slice of a CON rat. (**B**) Photomicrograph depicting retrogradely labeled neurons in V1 and V2 of the rat shown in (A). Inset, labeled V1 neurons displayed at higher magnification. (**C**) Diagram depicting the number and relative position of retrogradely labeled neurons in V1 and V2 of three CON rats (stars, circles, triangles). (**D**) Photomicrograph depicting FG spreading from the injection site (arrow) over all S1 layers in a 100 μm-thick coronal slice of a NWT rat. (**E**) Photomicrograph depicting retrogradely labeled neurons in V1 and V2 of the rat shown in (D). Inset, labeled V1 neurons displayed at higher magnification. Note the low density of stained neurons over all cortical layers. (**F**) Diagram depicting the number and relative position of retrogradely labeled neurons in V1 and V2 of three NWT rats (stars, circles, triangles). The underlying data of this figure can be found in S1 Data. http://dx.doi.org/10.6084/m9.figshare.1540797

To identify the substrate of altered multisensory processing after neonatal tactile restriction, we investigated the structural and functional communication between V1 and S1 of CON and NWT rats. We firstly examined the direct visual-somatosensory intrahemispheric projections in NWT (*n* = 5) and CON rats (*n* = 7) by injecting small amounts of the retrograde tracer Fluorogold (FG) into their S1 (Fig 4). Bright fluorescent back-labeled parental cell bodies in V1 feed-forwardly projecting to the ipsilateral barrel field were detected both in NWT and CON rats, indicating that intrahemispheric visual-somatosensory projections emerge even in the absence of unimodal stimuli during neonatal development. However, the maximal density of retrogradely labeled neurons was significantly (*p* = 0.023) lower (3–26 cells/0.16 mm^2^; mean: 8.6 ± 4.5 cells/0.16 mm^2^) in the V1 of NWT rats when compared to CON rats (11–56 cells/0.16 mm^2^; mean: 28.6 ± 6.4 cells/0.16 mm^2^) (S1D Table, S4 Fig). Thus, unimodal restriction during neonatal development led to a “sparsification” of the direct connectivity between S1 and V1.

**Fig 5:**
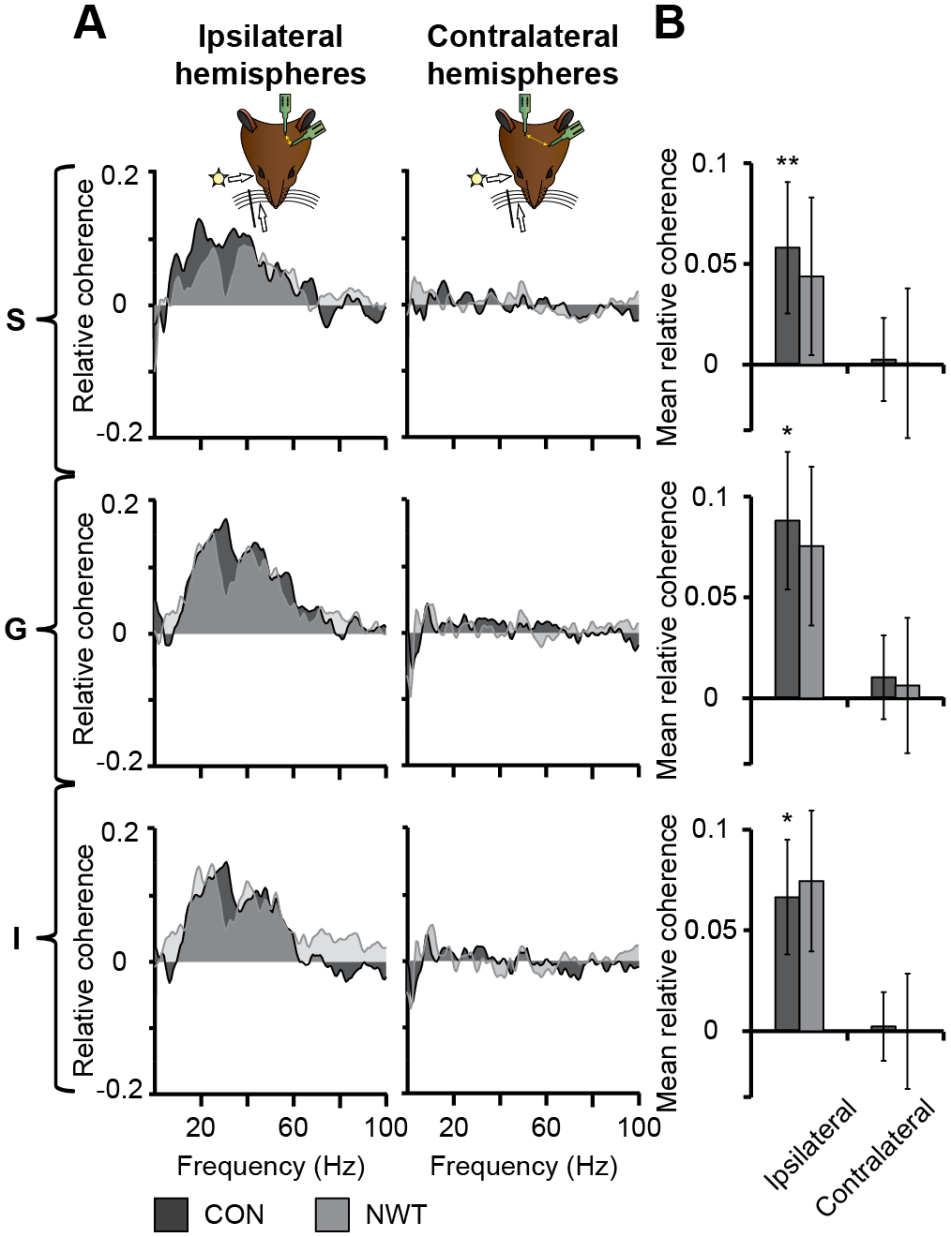
Coupling by synchrony and functional communication between S1 and V1 of CON and NWT rats. (**A**) Plots displaying the relative intra- (left) and interhemispheric (right) coherence change after bimodal stimulation between network activity in S (top), G (middle), and I (bottom) layers of the S1 and V1 of CON (black) and NWT rats (gray) in relationship to the frequency. The relative coherence was calculated by normalizing the values in all conditions to baseline (pre-stimulus) and subtracting unimodal coherence from the bimodal coherence. (**B**) Bar diagrams displaying the mean relative coherence between the layers of ipsi- and contralateral S1 and V1 when averaged for CON (black) and NWT rats (gray) in the frequency range of maximal coherence change (12–80 Hz). Significance values (*p* < 0.05 [*], *p* < 0.01 [**]) correspond to the comparison between uni- and bimodal stimulation conditions. The underlying data of this figure can be found in S1 Data. http://dx.doi.org/10.6084/m9.figshare.1540797

To test whether this reduced cortico-cortical connectivity after neonatal tactile restriction is accompanied by an impairment of the functional communication between primary sensory cortices, we assessed the impact of bimodal versus unimodal stimulation on the network synchrony between V1 and S1 of NWT (*n* = 9) and CON rats (*n* = 10) (Fig 5). The mean values of relative coherence were separately calculated for bimodal and unimodal stimulation conditions and their difference was averaged over 12–80 Hz (S5 Fig). In CON rats, bimodal stimulation significantly increased the intrahemispheric coherence between all layers of S1 and V1 by 0.058 ± 0.033, *p* = 0.008 (S), 0.088 ± 0.034, *p* = 0.017 (G), and 0.067 ± 0.03, *p* = 0.03 (I) when compared to unimodal stimulation (Fig 5A). The interhemispheric S1-V1 synchrony after bimodal versus unimodal stimulation was similarly affected in NWT rats (Fig 5B, S1E Table), even if their augmented coherence was below the significance threshold (S: 0.043 ± 0.040, *p* = 0.469; G: 0.074 ± 0.040, *p* = 0.12; I: 0.073 ± 0.035, *p* = 0.095).

**Fig 6:**
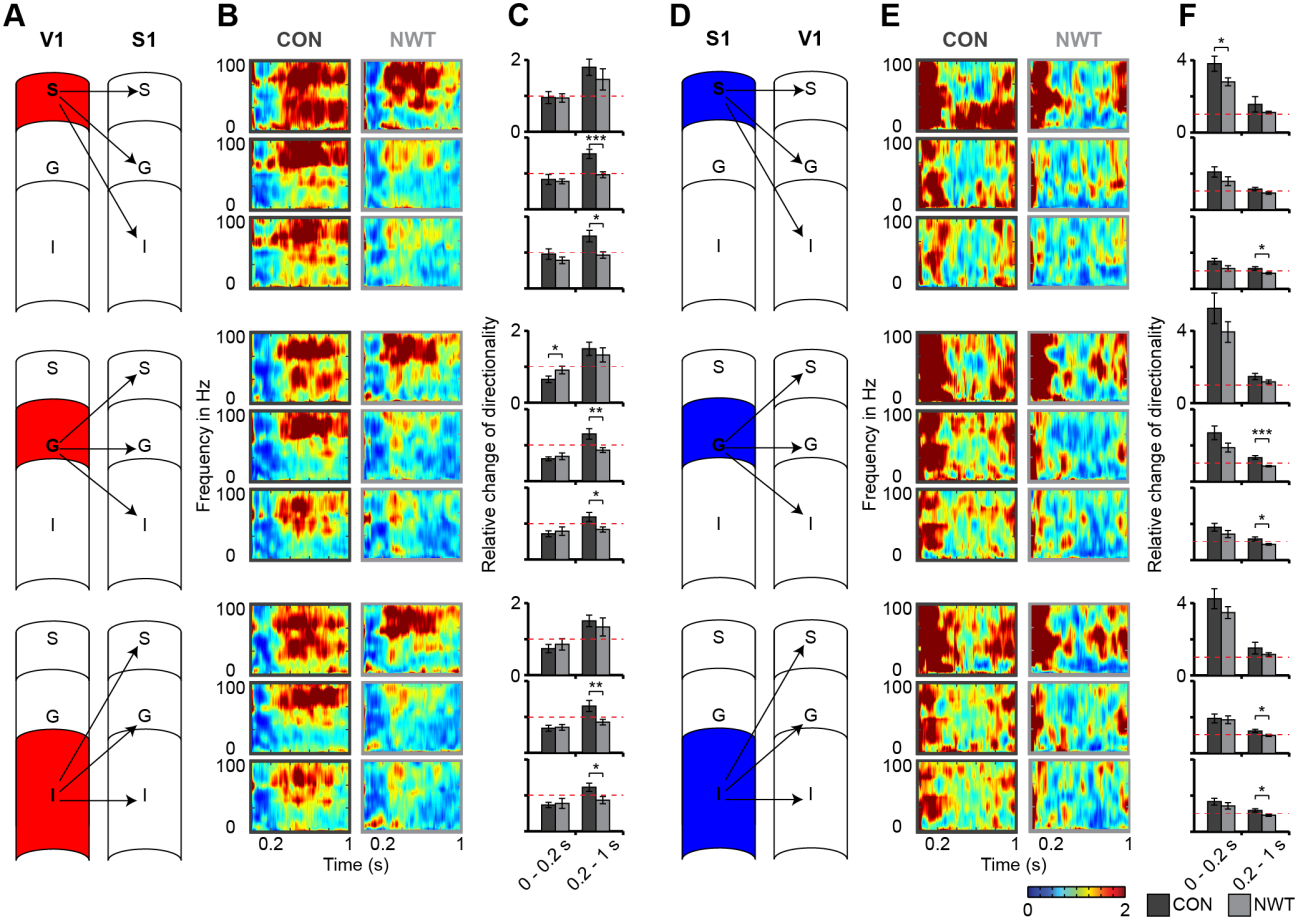
Directed interactions between S1 and V1 in CON and NWT rats. (**A**) Schematic representation of the analyzed direction of interaction from V1 to S1 layers. (**B**) Time- and frequency-resolved color-coded plots of Granger causality for cross-modal interactions of S (top), G (middle), and I (bottom) layers of the V1 with all S1 layers of CON (left) and NWT rats (right). The panels correspond to the interactions between different layers displayed in (A). In all panels, an increased drive compared to baseline (pre-stimulus) is encoded in red, whereas a decreased drive is encoded in blue. (**C**) Bar diagrams displaying the relative changes of directionality between V1 and S1 of CON (black) and NWT rats (gray) when averaged for the first 200 ms and the subsequent 800 ms after stimulus. Red dashed lines indicate the level of baseline causality. Significance values (*p* < 0.05 [*]) correspond to the comparison between CON and NWT rats. (**D**) Same as (A) for the directed interactions from S1 to V1. (**E**) Same as (B) for the directed interactions from S1 to V1. (**F**) Same as (C) for the directed interactions from S1 to V1. The underlying data of this figure can be found in S1 Data. http://dx.doi.org/10.6084/m9.figshare.1540797

The symmetric interdependence of coherence precluded reliable insights into the directionality of precisely timed interactions between S1 and V1. To test for causal information flow between primary sensory cortices in adult rats with and without neonatal tactile restriction, we performed Granger analysis for the V1 and S1 activity and plotted the directionality changes after bimodal versus unimodal stimulation (blue color in time-frequency plot: weaker directionality; red color in time-frequency plot: stronger directionality) (Figs 6 and S6). In CON rats (*n* = 10), the bimodal stimulation modulated the directed interactions between the primary sensory cortices by decreasing the drive from V1 to S1 during the first 200 ms post-stimulus and increasing it afterwards (Fig 6A–6C). In these rats, the drive from S1 to V1 was equally modulated by bimodal stimulation, yet the communication increased after stimulus, the strongest effect being detected within the time-window 0–200 ms post-stimulus (Fig 6D–6F, S1F Table). This cross-modal modulation of directed interactions V1 → S1 and S1 → V1 was decreased in NWT rats. The observed changes were particularly strong 200 to 1,000 ms after the stimulus in S and G layers (Fig 6B, 6C, 6E and 6F). These results indicate that transient tactile restriction during neonatal development decreased the functional communication between S1 and V1 in line with the sparsification of monosynaptic cortico-cortical projections.

### Unimodal Experience during Neonatal Development Is Mandatory for the Maturation of Cross-Modal Matching Abilities of Object Features

To assess the behavioral correlate of impaired multisensory processing at the neural level after transient neonatal tactile restriction, we tested CON (*n* = 51) and NWT rats (*n* = 51) in a modified cross-modal novel object recognition task (NOR) [31]. This task uses the intrinsic preference of rodents for novel objects [32]. Rats were allowed to explore two identical objects during the sample phase. Subsequently, one of these two identical objects was replaced by a novel object with different features during the choice phase. The paradigm was conducted under four sensory conditions (S7 Fig). We allowed (i) simultaneous visual and tactile exploration (bimodal condition), (ii) only tactile exploration (tactile condition, i.e., the experiment was performed under red light to prevent visual exploration) or (iii) only visual exploration (visual condition, i.e., objects were placed under familiar glass containers to prevent tactile interactions). The fourth condition was used to test whether rats could match tactile with visual information (cross-modal condition). For this, the sensory modality used for object exploration differed between the sample and the choice phase. Rats needed to transfer the cross-modal information between modalities to recognize the novel object. These four conditions were tested in two experimental settings. In the first setting, termed as continuous setting, each rat was consecutively assigned to the bimodal, one unimodal and the cross-modal conditions. In contrast, in the second setting, termed as discrete setting, each rat was assigned to only one condition (S7 Fig, S2 Table). The familiarization phase was identical for both settings.

**Fig 7:**
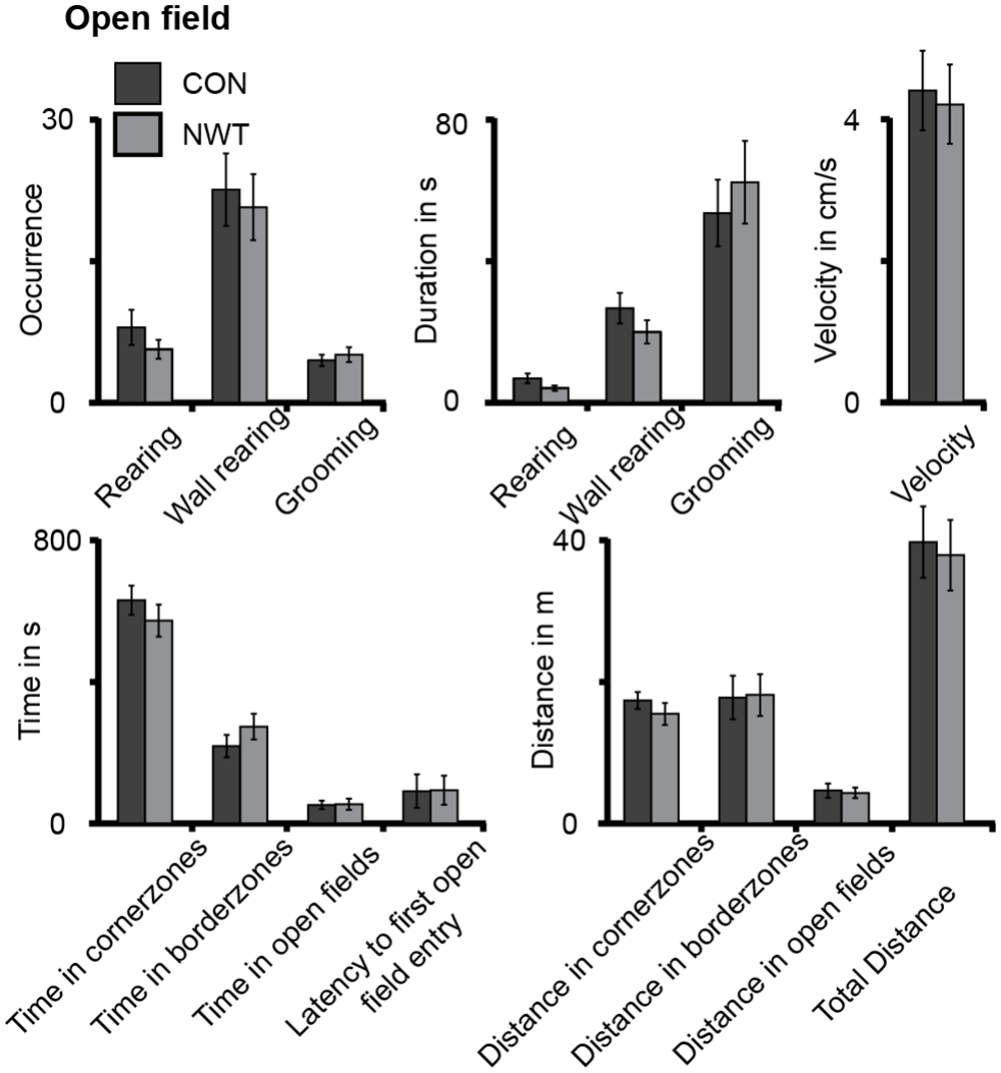
Exploratory and anxiety behavior of CON and NWT rats. Bar diagrams displaying the time spent and latency to enter as well as the distance travelled in each zone of the open field arena, the travel velocity, the mean frequency and duration of rearing, wall rearing, and grooming events averaged for 15 CON (black) and 18 NWT rats (gray). The underlying data of this figure can be found in S1 Data. http://dx.doi.org/10.6084/m9.figshare.1540797

Since in an open field pre-test the travelled distance, speed of locomotion, the duration and occurrence of rearing, wall-rearing, and grooming, as well as the time spent in the different areas of the open field was similar for CON and NWT rats, differences in locomotor and anxiety behavior between groups were excluded (Fig 7, S1G Table).

**Fig 8:**
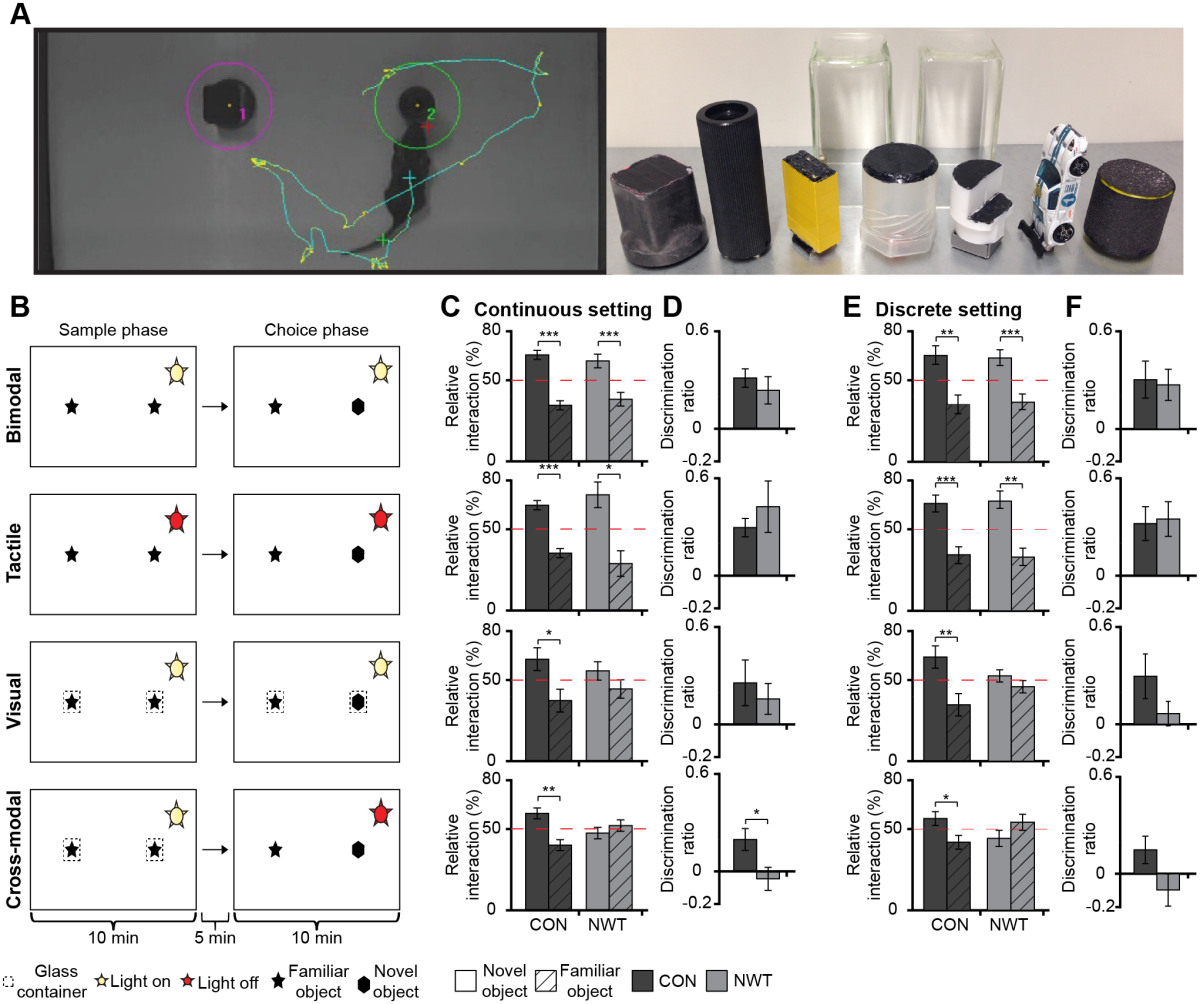
Impact of neonatal tactile restriction on the adult cross-modal matching performance. (**A**) Photograph displaying the tracking of a rat exploring two objects during NOR task (left) as well as the glass containers and objects with different shapes and textures that were used for the sample and choice phases of NOR (right). The red, cyan, and green crosses mark the head, center of gravity, and the tail of rat, respectively. (**B**) Illustration of the four experimental paradigms for testing novelty recognition and cross-modal matching. In the continuous setting each rat was consecutively tested in each experimental paradigm. In the discrete setting each rat was tested in a single paradigm (i.e., either bimodal, tactile, visual, cross-modal). (**C**) Bar diagrams displaying the relative interaction time spent by 15 CON (black) and 18 NWT (gray) rats with the new versus familiar object when investigated in the continuous setting during the experimental paradigms illustrated in (B). The red dotted line indicates chance level. (**D**) Bar diagrams displaying the discrimination ratios of new versus familiar objects during the experimental paradigm illustrated in (B) for CON (black) and NWT rats (gray) tested in the continuous setting. (**E**) Same as (C) for 36 CON and 33 NWT rats when investigated in the discrete setting (*n* = 9 CON, 10 NWT in bimodal testing, *n* = 10 CON, 9 NWT in tactile testing, *n* = 8 CON, 6 NWT in visual testing and *n* = 9 CON, 8 NWT in cross-modal testing). (**F**) Bar diagrams displaying the discrimination ratios of new versus familiar objects during the experimental paradigm illustrated in (B) for CON (black) and NWT rats (gray) tested in the discrete setting. The underlying data of this figure can be found in S1 Data. http://dx.doi.org/10.6084/m9.figshare.1540797

In both settings, CON and NWT rats spent more time with the exploration of the new object compared to the time spent with the exploration of the familiar object when they relied on both senses (continuous setting, CON: 65.5 ± 2.8% of the total time, *p* < 0.001; NWT: 61.8 ± 4.2%, *p* < 0.001; discrete setting, CON: 65.1 ± 5.7% of the total time, p = 0.002; NWT: 63.5 ± 4.8%, *p* = 0.001) (Fig 8). Similarly, the rats relying on tactile perception explored the novel object longer (continuous setting, CON: 64.8 ± 2.8% of the total time, *p* < 0.001; NWT: 71.1 ± 7.9%, *p* < 0.001; discrete setting, CON: 65.7 ± 5.2% of the total time, *p* < 0.001; NWT: 67.1 ± 5.3%, *p* = 0.009). In both groups of rats the visual recognition of the novel object was less precise than the tactile recognition and this was independent of the experimental settings (continuous setting, CON: 62.7 ± 7.0% of the total time, *p* = 0.025; NWT: 55.6 ± 5.6%, *p* = 0.18; discrete setting, CON: 64.94 ± 7.0% of the total time, *p* = 0.009; NWT: 53.39 ± 3.84%, *p* = 0.241). For bimodal, tactile and visual conditions, the discrimination ratio did not differ between groups and experimental settings. Under cross-modal conditions, CON rats spent significantly longer time exploring the novel object (continuous setting, 59.7 ± 3.3% of the total time, *p* = 0.002; discrete setting, 57.4 ± 4.3% of the total time, *p* = 0.027), whereas NWT rats failed recognizing the novel object (continuous setting, 47.7 ± 3.5%, *p* = 0.37; discrete setting, 44.97 ± 5.03% of the total time, *p* = 0.179). In only this matching test, the recognition performance of NWT rats was lower when compared to CON rats (Figs 8 and S1H). These results indicate that transient tactile restriction during neonatal development permanently impairs cross-modal matching abilities, while leaving the unimodal object recognition largely unaffected.

## Discussion

Cross-modal modulation of neuronal assemblies in primary sensory cortices is an important mechanism of multisensory processing in adult rodents [8] and across much of the brain in more complex species. To which degree the development of this mechanism depends on uni- or cross-modal experience is still unresolved. The present study provides first insights into the essential requirements of the neural basis of multisensory development. We demonstrate that unimodal experience during neonatal development (i) refines the somatosensory topography and tactile processing in S1 (Figs 1 and 2), (ii) shapes visual-tactile interactions in S1 and along the subcortico-cortical sensory tract (Fig 3), (iii) ensures the correct maturation of direct cortico-cortical projections (Fig 4), (iv) enables multisensory communication by synchrony and modulation of directed interactions within adult cortico-cortical networks (Figs 5 and 6), and (v) is necessary for the development of cross-modal matching abilities (Figs 7 and 8). These findings point towards a sensitive/critical period in the multisensory maturation of both neuronal networks and behavior.

Unimodal input and thus, experience-driven electrical activity, has been shown to shape and refine the structure and function of cortical circuits [23,33]. For example, monocular deprivation during development dramatically alters the V1 topography, visually induced responses, and visual acuity of the deprived eye [34,35]. Similarly, tactile deprivation of the vibrissal sensory system by permanent damage of the sensory periphery (cauterization or lesion of follicle sinuses) causes extensive structural changes in S1 [36]. Besides this irreversible and complete tactile deprivation, even transient restriction of tactile inputs in neonatal rats by daily whisker trimming seems to affect the morphology of barrels. In line with the increased dendritic span and spine density of spiny stellate neurons in layer IV after transient vibrissal deprivation [27,37], the size of the barrels in NWT rats was slightly, yet not significantly, increased (Fig 2). During neonatal development, the transient absence of unimodal inputs may decrease the global level of activation within neonatal S1, since it diminishes non-whisking related (so-called spontaneous) patterns of oscillatory activity and the individual neuronal firing [18,38,39]. The changed timing of individual spiking, which most likely resulted from the reduced spontaneous activity, perturbed the developmental Hebb-like processes that control the refinement of projections [40]. Thus, the absence of unisensory experience during a defined developmental time window may have long-lasting structural and functional effects on unisensory processing. At adulthood, tactile EPs were enhanced in NWT rats (Fig 2). This increase in amplitude correlated with the enlarged excitatory receptive fields and augmented cellular responses in layer IV [41]. Since the responses of neurons in layer II/III were normal [42], this altered evoked activity most likely reflects an impairment of thalamo-cortical responses. Moreover, the neonatal tactile restriction has been reported to cause permanent behavioral deficits in texture discrimination or gap crossing tests [27,43]. In contrast, we did not observe significant deficits in tactile object recognition skills (Fig 8). This discrepancy may be explained by the lower sensorimotor complexity of the present matching task.

In addition to the deficits in unisensory processing, the transient tactile restriction during neonatal development caused reduced multisensory processing in S1 (Fig 3). In contrast to the effects observed in CON rats, neither the EPs were supra-additively enhanced nor the induced activity was modulated by a cross-modal stimulus in NWT rats. It might be argued that the absence of a multisensory enhancement resulted from the effects of neonatal tactile restriction on unisensory processing. As a result, an additional visual stimulus was not able to further augment the enlarged tactile responses in NWT rats. However, a multisensory EP enhancement was not only absent in the G layers but additionally in the S layers, where the unisensory processing was not augmented by neonatal restriction. Thus, we alternatively suggest that the absence of a multisensory enhancement in NWT rats was due to the fact that the transient unisensory deprivation affected not only the cortico-cortical processing, but also the feed-forward thalamo-cortical interactions. We have previously shown that the cross-modal augmentation of early (~30 ms) evoked responses in S1 results from the integration of sensory inputs along the sensory tract preceding primary cortices, most likely within thalamic nuclei [8]. The absence of a cross-modal EP enhancement suggests that unisensory experience controls the emergence of multisensory integration at the subcortical level. The mechanisms by which neonatal unimodal inputs shape the physiology of individual thalamic neurons [44] and the axonal patterning along the feed-forward sensory tract remain to be elucidated.

Most research of the past has investigated the emergence of multisensory processing in the SC and association cortices. Multisensory interaction in these areas has been demonstrated to depend on sensory experience [25]. When animals were reared in the dark [45,46] or in omnidirectional sound conditions [47], SC neurons did not integrate any visual-nonvisual or auditory-nonauditory cue pairs, respectively. Critically, these deprivation paradigms precluded not only unisensory but additionally concurrent experience with multiple modalities. In contrast, in the present experimental paradigm we partially deprived the animals of tactile experience during an early neonatal time period when visual inputs activate neither the sensory periphery nor subcortical and cortical circuits (Fig 1). The pigmented rats opened their eyes at P16–17 and the first visual evoked potentials in V1 emerged 2–3 d before eye opening, reflecting the onset of retinal light sensitivity [48]. By this time, the length and function of neonatally-trimmed whiskers were already fully restored in NWT rats. Thus, the reduction of tactile input during early development caused the multisensory deficits in NWT rats. Though the alternative explanation that impairment of unisensory processing may contribute to multisensory deficits cannot be fully excluded, the intact tactile object recognition of NWT rats is inconsistent with this explanation. Whether distinct intracortical circuits differently involving the S, G, and I layers mechanistically disconnect uni- and multisensory processing remains to be investigated.

The present study identified both structural and functional deficits within neocortical networks that might cause abnormal cross-modal processing after neonatal unisensory restriction. Deprivation of a sensory modality has been reported to cause the reorganization of sensory systems for another modality (cross-modal plasticity) [49,50]. For instance, permanent visual deprivation in neonatal hamsters or mice resulted in reciprocal connections between V1 and other brain regions, such as the major midbrain auditory nucleus, the inferior colliculus and S1 [51,52]. Consequently, V1 neurons responded to tactile inputs by increasing their firing rate and synchronizing their depolarization [51,53]. It has been hypothesized that these plastic effects are mediated by the stronger reliance on the remaining modality after deprivation [50,54]. However, the present findings showed that transient unisensory restriction during neonatal development led to reduced rather than an exuberant V1-S1 connectivity (Fig 4). In line with these results, visual inputs did not evoke an increased response in S1 in NWT rats. Fewer projections coupling the primary sensory cortices of NWT rats may have caused a phase reset in an atypical, presumably suboptimal frequency band (Fig 3). Setting of the oscillatory activity to a non-random instantaneous phase has been identified as a ubiquitous mechanism for increasing the processing efficiency of individual stimuli [55,56]. A cross-modal phase reset might improve the predictability of the associated stimulus or cause an enhanced salience of the cross-modal stimulus, as proposed for visual-auditory interactions [57]. A phase rest in a different frequency band, as observed in NWT rats, might either has prevented an efficient perceptual encoding or has caused a lower stimulus salience. Consequently, we observed a lower cross-modal transfer during object recognition task in NWT rats. Besides influencing the phase of ongoing oscillations, the fewer cortico-cortical projections seem to decrease the power, synchrony and drive between V1 and S1 in NWT rats (Figs 5 and 6).

The present data provide evidence for the critical role of neonatal unimodal experience for the establishment of multisensory interplay both at the neural and behavioral level. Rats have previously been shown to own excellent skills for cross-modal object recognition [31]. Our data confirmed these findings and identified unimodal inputs during development as a critical factor for the emergence of cross-modal matching abilities of object features (Fig 8). Rats experiencing neonatal tactile restriction were not able to use previously acquired tactile information for recognizing visually explored objects and vice versa. Remarkably, the behavioral changes were very robust and replicated under different experimental settings. This suggests that learning effects did not influence the behavioral outcome and that the divergent features of objects used for testing did not allow unspecific familiarization.

In contrast to the reduced multisensory abilities, the unisensory object recognition was intact after neonatal restriction. In both groups of rats visual discrimination of objects was worse than tactile object discrimination. One explanation for this finding might be the weak saliency or contrast of the used objects or a too small distance to the objects. The abilities for cross-modal object recognition seem to critically depend not only on unimodal sensory inputs during defined developmental stage but also on the integrity of the perirhinal and posterior parietal cortices important for memory retention and retrieval [31]. The precise neural mechanisms integrating sensory and mnemonic aspects of the task remain to be elucidated.

In conclusion, the present findings demonstrate that the absence of adequate unisensory experience during a specific developmental period impairs the setting up of cortico-cortical and cortico-subcortical connectivity as well as functional network communication underlying multisensory behavior.

## Materials and Methods

All experiments were performed in compliance with the German laws and the guidelines of the European Community for the use of animals in research and were approved by the local ethical committee (No. 21/10, 95/15).

### Electrophysiological Recordings

Extracellular recordings were performed under light urethane anesthesia (0.5 g/kg, i.p., Sigma-Aldrich, St. Louis, MO) as previously described [8]. One-shank 16-channel electrodes with 100 μm spacing between recording sites (0.5–3 MΩ, Silicon Michigan probes, NeuroNexus Technologies, Ann Arbor, MI) were perpendicularly inserted into S1 (2.4–2.6 mm posterior and 5.5–5.8 mm lateral to bregma) and V1 (6.9–7.1 mm posterior and 3.4–3.7 mm lateral to bregma) bilaterally to a depth of 1.6 mm. Electrodes were labeled with DiI (1,1′-dioctadecyl-3,3,3′,3′-tetramethyl indocarbocyanine, Invitrogen, Carlsbad, CA) to enable the post-mortem reconstruction of electrode tracks in histological sections. A custom-built stimulation device placed in front of the animal was used to achieve unimodal (light-flash, whisker deflection) or bimodal stimulation [8]. During bimodal stimulation, whisker deflection and light-flashes (50 ms, 300 lx, full eye field stimulation) were presented simultaneously in the same (congruent) hemifields with respect to the tactile stimulus. During tactile stimulation, all whiskers were displaced by an upward moving stick activated through compressed air-controlled roundline cylinders gated via solenoid valves. The stimulation device produced almost silent, nonelectrical stimulation with precise timing (0.013 ± 0.810 ms) that was constant over all trials/ conditions. The stimulation device randomized stimuli in the different stimulation conditions (unimodal visual, unimodal tactile, bimodal). The non-stimulated eye was covered with an aluminum foil patch and ears were sealed with cotton to avoid auditory inputs. The solenoid valves were placed outside the setup and isolated with foamed material to lower their noise. For all stimulation conditions this noise was constant, running out a possible contribution of auditory modulation. Each type of stimulus was presented 100 ± 10 times with an inter-stimulus-interval of 6.5 ± 0.5 s.

### Neonatal Trimming of Whiskers and Somatic Development

The transient restriction of neonatal vibrissal input was achieved by cutting the whiskers close to the intersection with the skin. All of the macrovibrissae were daily trimmed to <0.4 mm from 3–12 h after birth until P6 with an electric micro shaver (ChroMini, Moser, Unterkirnach, Germany). Rats were daily weighed and whisker length was measured via a caliper gauge under the microscope (20–40x magnification) (SZX51, Olympus, Tokyo, Japan). The onset of adult whisking behavior (amplitude >45°, frequency >5 Hz, duration >2 s) and time of eye opening were detected.

### Retrograde Tracing and Histology

For retrograde tracing, anesthetized rats were fixed in the stereotaxic apparatus on a custom-built mold and received unilateral injections of Fluorogold (Fluorochrome, Denver, CO) in S1 (2.4–2.6 mm posterior and 5.5–5.8 mm lateral to bregma). A total volume of 100 nl FG (5% in PBS, 30 nl/min) was delivered via a 26G needle attached to a pump controller (Micro4, WPI, Sarasota, FL). Three to five days after FG injection, animals were deeply anesthetized with ketamine/xylazine and perfused transcardially with 4% paraformaldehyde (PFA). Brains were removed and postfixed in 4% PFA for 24–72 h. Blocks of tissue containing S1 or V1 were sectioned in the coronal plane at 100 μm, air dried, and examined using ultraviolet excitation filter of the fluorescence microscope (SZX16, Digital camera DP72, Olympus, Tokyo, Japan). For quantification, FG-stained cells were detected (cellSens 1.4.1, Olympus) and counted by eye in an area of 0.16 mm^2^ in S1 (S4 Fig).

For COX staining, the rats were transcardially perfused with 4% PFA immediately after electrophysiological recordings. Their brains were removed and halved along the midline. Subcortical brain regions were removed, the cortex was flattened between two acrylic glass plates and postfixed in 4% PFA for 24–72 h. The flattened cortices were sectioned in the transverse plane at 100 μm and processed for COX histochemistry (Fig 2A). The sections were incubated in a solution containing diaminobenzidine (0.5 mg/ml), cytochrome C (0.6 mg/ml), katalase (0.36 mg/ml), saccharose (44.4 mg/ml), and examined using light microscopy. Individual barrels were visually identified and their borders manually defined by monitoring the contrast changes (cellSens 1.4.1 and Adobe Photoshop, 11.0.2). The size of the first four barrels in the rows A–D was calculated and compared between CON and NWT rats (S2B Table).

### Behavioral Testing

The litters used for behavioral testing included both males and females to avoid sex-based maternal behavior biases [58]. All behavioral tests were conducted during the light phase and by one investigator.

Rats were daily weighed from P0 to P21. All experiments were conducted in a black acrylic glass arena with a white ground (L: 52 cm, W: 30 cm, H: 32 cm). The seven objects used for testing of novelty recognition were seven differently shaped and textured, easy-to-clean items (Fig 8A, S2A Table) that were provided with magnets to fix them to the bottom of the arena (15 cm from the borders and 10.5 cm from the center of the arena). Object sizes (H: 5–9 cm, diameter: 3–4.5 cm) were smaller than twice the size of the rat and did not resemble living stimuli (no eye spots, predator shape). Glass containers (L: 6 cm, W: 5.5 cm, H: 10.5 cm) were placed over the objects to prevent tactile object exploration during visual exploration. During tactile exploration, the light was switched off and a red-tinted bulb (18 W) illuminated the arena to exclude any contribution of visual perception to the exploration of objects. After every experiment the objects and arena were cleaned with 70% ethanol to remove all odors. A black and white CCD camera (Videor Technical E. Hartig GmbH, Roedermark, Germany) was mounted 90 cm above the arena and connected to a PC via PCI interface serving as frame grabber for video tracking software (Video Mot2 software, TSE Systems GmbH, Bad Homburg, Germany).

First, exploratory behavior was assessed 1–3 d after eye opening in P17–19 rats. The animals were allowed to freely explore the empty arena for 15 min. During this time, which was defined as habituation phase (open field), rearing, wall-rearing and grooming were quantified in their occurrence and duration. The floor of the arena was digitally subdivided into 16 zones of the same size (four in the corners, eight at the borders, and four in the open field). The time spent in, the travel speed and distance were measured for each zone.

Second, rats were familiarized with the arena and testing conditions. This phase was defined as familiarization phase (S7 Fig). Five minutes after the open-field test, P17–19 rats were placed back into the arena and allowed to explore for 5 min the two glass containers that later were used in the recognition task exclusively relying on visual perception. The following day, rats were familiarized again for 5 min in the arena with the glass containers and for 10 min with the red light-illuminated arena and glass containers.

Third, novel object recognition was investigated in a series of tests. All protocols for assessing bimodal, unimodal and cross-modal object recognition were tested in P19–23 rats. During the sample trials, rats were placed into an arena containing two identical objects and released against the center of the opposite wall with their back to the objects. After 10 min of free exploration of objects, the rat was returned to a temporary holding cage. During the test trials one familiar object was replaced by a novel object with a different texture and visual appearance (e.g., cube instead of cylinder). One day after familiarization with the empty arena and the two glass containers, two groups of rats were tested in different behavioral settings. In the first setting (continuous setting), bimodal object recognition was tested in rats relying on both visual and tactile perception. Two days after familiarization, unimodal object recognition was tested in rats randomly assigned to either the group relying exclusively on visual perception (light, objects covered by glass containers) or the group relying exclusively on tactile perception (red light, no glass containers). Three days after familiarization, cross-modal object recognition (cross-modal matching) was tested in rats relying in the choice phase on a different sensory modality than during the sample phase. In the second behavioral setting (discrete setting), each rat was tested in only one sensory condition.

The trials were video-tracked and the analysis was performed using the Video Mot2 analysis software. The object recognition module of the software was used and a three-point tracking method identified the head, the rear and the center of gravity of the rat. Rats that did not interact with objects in the sample phase were excluded from analysis. Digitally, a circular zone of 2 cm was created around each object and every entry of the head point into this area was considered as object interaction. For tactile and bimodal exploration, the first 5 min of interaction with the objects were analyzed, whereas for visual exploration, the analysis was confined to the first 2 min. Climbing or sitting on the object, mirrored by the presence of both head and center of gravity points within the circular zone, were not counted as interactions. Relative interaction time t_RI_ was calculated by

$${\text{t}}_{\text{RI}}=\;\frac{{\text{t}}_{\text{NO}}}{{\text{t}}_{\text{NO}}+{\text{t}}_{\text{FO}}}$$

where t_NO_/t_FO_ is the time that the rat spent with the novel/familiar object. Significance was tested between objects. Discrimination ratio DR was calculated as

$$\text{DR}=\;\frac{{\text{t}}_{\text{NO}}-{\text{t}}_{\text{FO}}}{{\text{t}}_{\text{NO}}+{\text{t}}_{\text{FO}}}$$

### Data Analysis

Data were imported and analyzed offline using custom-written tools in Matlab software version 7.7 (MathWorks, Natick, MA). Data were recorded at a sampling rate of 32 kHz. For anti-aliasing, the signal was band-pass filtered (0.1 Hz–5 kHz) by the Neuralynx recording system. A third order Butterworth filter was applied. The subsequent downsampling of the data was analysis-dependent (factor 5 for EP calculation, factor 30 for spectral and phase analysis, factor 10 for coherence analysis, factor 128 for Granger directionality analysis).

#### Calculation of evoked potentials

Continuous recordings were epoched offline and 1 s-long time windows (300 ms pre- and 700 ms post-stimulus) corresponding to each stimulation condition (unimodal visual, unimodal tactile, bimodal) were averaged for each recording. Epochs with stimulation artifacts or offsets (i.e., absolute value of the mean potential during 1 trial>200 μV) were removed. The properties of averaged EPs and the tracks of DiI-labeled electrode were used to confirm the recording positions in every rat. Current source density (CSD) analysis allowed functional identification of S, G, and I layers at higher spatial resolution (Fig 2C). One-dimensional CSD profiles were calculated to a five point formula. The CSD values I_m_ were derived from the smoothed second spatial derivative of the extracellular field potentials Φ and calculated by

$${\text{I}}_{\text{m}}=\;-\frac{1}{{\text{kh}}^{2}}\sum_{\text{m}=-\text{n}}^{\text{n}}{\text{a}}_{\text{m}}\Phi \left(\text{r}+\text{mh}\right)$$

where h is the distance between electrodes (100 μm) and r is the coordinate perpendicular to the cortical layer (n = 2, k = 7 a_0_ = -2, a _± 1_ = -1 and a _± 2_ = 2). The number of time windows averaged over rats for each recording site was kept constant across stimulation conditions. EP peaks were detected as local LFP maxima/minima within sliding time windows of 100 ms and were defined as positive (P) or negative (N) based on surface polarity (i.e., polarity in the upper layers). Their amplitudes were averaged over all trials and animals, tested for significant differences between conditions and corrected with the Holm-Bonferroni method. After the alignment of electrode position between animals and artifact/offset removal, the resulting number of animals and trials was for CON rats S layer: nine rats (14 hemispheres), 1,256 trials with a stimulation range of 44–100 trials/rat; G layer: ten rats (19 hemispheres), 1,754 trials with a stimulation range of 47–100 trials/rat; I layer: nine rats (16 hemispheres), 1,462 trials with a stimulation range of 47–100 trials/rat and for the NWT rats S layer: eight rats (12 hemispheres), 1,089 trials with a stimulation range of 55–100 trials/rat; G layer: nine rats (18 hemispheres), 1,617 trials with a stimulation range of 62–100 trials/rat; I layer: nine rats (14 hemispheres), 1,315 trials with a stimulation range of 80–100 trials/rat.

#### Spectral analysis of induced activity

For each stimulation trial, continuous wavelet coefficients C were calculated for 1,500 ms time windows (500 ms pre- and 1,000 ms post-stimulus) at frequency scale a and position b by

$${\text{C}}_{\text{a},\text{b}}=\underset{\text{R}}{\int }\Phi \left(\text{t}\right)\frac{1}{\sqrt{\text{a}}}\bar{\text{Ψ}\left(\frac{\text{t}-\text{b}}{\text{a}}\right)}\text{dt}$$

where Ψ is a Morlet wavelet, defined as

$$\text{Ψ}\left(\text{x}\right)=e^{\left(-\frac{x^{2}}{2}\right)}\text{cos}\left(5x\right)\;t\in \left(-\infty ,+\infty \right)$$

They were corrected for pink noise by normalization to the coefficients of baseline activity at every frequency (i.e., dividing every coefficient by the temporal mean of the baseline 100–300 ms pre-stimulus). The timing of power modulation is less precise because of the pink noise correction and low time resolution of wavelets in low frequency range. Baseline normalized wavelets were averaged for all rats in all stimulation conditions and their coefficients were tested for significant differences between unimodal and bimodal conditions (S1A Fig). Significant coefficients were plotted and clusters (>50 points) were grouped in time-frequency windows (I: 145–228 ms/30–100 Hz, II: 447–545 ms/30–100 Hz, III: 854–903 ms/30–100 Hz) (S1B Fig). In these windows, the mean relative power change compared to baseline was calculated for every animal (S1C and S1D Fig). For a better comparison between conditions, we computed the difference between bimodal and unimodal mean relative power for every rat and tested for significance between conditions (S1E Fig). Furthermore, mean relative power difference was tested for significance between NWT and CON rats.

#### Phase analysis

Phase distribution across trials was characterized by calculating the mean length of the resulting vector. For this, single trial LFPs during 1,500 ms-long time windows (500 ms pre- and 1,000 ms post-stimulus) were filtered (third order Butterworth bandpass filter) in four frequency bands (theta: 4–8 Hz; alpha: 8–12 Hz; beta: 12–30 Hz; gamma: 30–100 Hz). The phase of oscillatory activity was extracted using the Hilbert transform at every time point and circular histograms for the phases of all trials were created (S3A and S3B Fig). The mean resultant phase vectors were determined (S3B and S3C Fig), the length of these vectors was extracted via circular statistical methods (Circular Statistic Toolbox) and baseline-normalized (e.g., temporal mean 300–500 ms pre-stimulus was subtracted). Due to zero-phase digital filtering, the phase was not distorted but the time resolution of phase distribution was poor. The 99% confidence intervals were calculated with a z-value corrected for the total number of time-points (*n* = 1628, z = 4.5214).

#### Coherence analysis

As spectral measure of correlation between two signals across frequency, the coherence was calculated from the cross-spectral density between the two signals and normalized by the power spectral density of each [59]. The computation of the magnitude-squared coherence C over frequency (f) was performed according to the formula

$$C_{xy}\left(f\right)=\;\frac{{\left|P_{xy}\left(f\right)\right|}^{2}}{P_{xx}\left(f\right)P_{yy}\left(f\right)}$$

where x and y are the combined signals of all trials of a rat in one stimulation condition in different layers of S1 and V1. P_xx_ and P_yy_ are the power spectral densities and P_xy_ is the cross power spectral density of the corresponding signals. With this method we calculated the post-stimulus coherence (0–1,000 ms post-stimulus) between the cortices in the different stimulation conditions (C_V_ = unimodal visual, C_T_ = unimodal tactile, C_B_ = bimodal). For calculating stimulus-induced coherence change, pre-stimulus coherences [0–1,000 ms pre-stimulus (C_PV_, C_PT_, C_PB_)] were subtracted from post-stimulus coherences (S5A and S5B Fig). The high coherence values obtained shortly after bimodal stimulation were not due to high synchronization between cortical areas, but resulted artificially, because the evoked responses in both cortices had a similar shape. To correct for this, we randomized single trial tactile-stimulation signals in S1 and calculated the coherence to randomized single trial visual-stimulation signals in V1 (C_VT_). The corrected cross-modal coherence-modification C_S_ (S5C Fig) was calculated by

$$C_{S}=(C_{B}-C_{PB})-\left(\left(C_{V}-C_{PV}\right)+\left(C_{T}-C_{PT}\right)+\left(C_{VT}-C_{PVT}\right)\right)$$

For quantification and statistics the mean values of C_S_ were calculated between 12–80 Hz for every rat, the frequency range with the most prominent coherence changes (S5D Fig). Coherence differences were calculated for intrahemispheric S1 and V1 (i.e., ipsilateral hemispheres) with contralateral stimulus as well as for interhemispheric S1 and V1 (i.e., contralateral hemispheres) with stimulus contralateral to S1.

#### Directionality

The analysis of directed interactions was based on the concepts of Wiener-Granger causality applied to autoregressive models of the data [60,61]. For two simultaneously measured time series, one series can be defined as causal to the other if the second series can be better predicted by incorporating past knowledge from the first one. To preprocess the data, calculate directionality values, and test the model, routines from the BSMART and GCCA Toolboxes were used [62,63]. Due to the rapidly changing (non-stationary) nature of evoked signals a method was used that decompose the signal into short time windows to treat it as approximately stationary [64]. First, the signals after bimodal stimulations both from 0 to 1,000 ms post-stimulus as well as 0 to 1,000 ms pre-stimulus were preprocessed by removing deterministic linear trends and single trial temporal mean as well as the ensemble mean (S6A Fig). Recordings that did not show covariance-stationarity in all time-series for any condition were excluded (Dickey-Fuller test for unit-roots). The best model order was calculated by Akaike and Bayesian Information Criterion (S6B Fig). Granger-causal influences I from j on i (signals in layers of V1 on signals in layers of S1 and vice versa) were defined as

$$I_{j\to i}\left(f\right)=-log\left(1-\frac{\left(V_{j,i}-\frac{V_{i,j}^{2}}{V_{j,i}}\right)|H_{i,j}\left(f\right){|}^{2}}{S_{j,i}\left(f\right)}\right)$$

where V(f) is the noise covariance, H(f) is the transfer function and S(f) is the spectral matrix of the bivariate model of X_i_ or X_j_, respectively. Small time windows (100 ms) were defined and moved over the complete time. The validity of the model in terms of consistency, residual noise correlation and stability was tested and only recordings fulfilling these criteria were considered (S6C–S6E Fig). To calculate the cross-modal modulation of causality, post-stimulus Granger effects were divided by the temporal mean of pre-stimulus effects at all frequencies. The mean relative change of directionality was calculated in a time window from 0–200 ms post-stimulus and from 200–1,000 ms post-stimulus and tested for significance between NWT and CON rats.

#### Multi-unit activity (MUA) analysis

The signal was high-pass filtered (>400 Hz) using a 3^rd^ Butterworth zero-phase filter. The detected signal was full-wave rectified by flipping negative values into the positive range and averaged for all trials and animals for a time window of 50 ms post-stimulus. The time point of maximal value of averaged signal was detected. For each condition and animal the signal amplitude at this time point was calculated, the resulting values being averaged and displayed as bar diagrams.

### Statistics

Data in the text are presented as mean ± SEM. All values were tested for normal distribution with Lilliefors test (α = 0.05). For normally distributed values *t* tests, for not normally distributed values or if n<8, Kruskal-Wallis test detecting significance levels of *p* < 0.05 (*),*p* < 0.01 (**), and *p* < 0.001 (***) were used (S1 Table). For behavioral testing and anatomical investigations the given *n* corresponds to the number of investigated rats. For statistics on EPs, the given *n* corresponds to the number of stimulation trials that reliably evoked activity with consistent peak timing. This better mirrors the subtle modulatory effects on EPs, since cross-modal effects were previously observed for congruent (i.e., whisker deflection and light stimulus were presented in the same hemifield) but not for incongruent (stimuli applied on opposite hemifields) stimulation conditions when tested in the same group of rats [8]. For the remaining electrophysiological data, we considered the values recorded from each hemisphere, because, according to our stimulation paradigm (Fig 1A), they were independent (different stimulation time-points, recording electrode, recording depth). Variable *n* values resulted from the exclusion of animals with insufficient behavioral performance during control conditions and of recordings outside the layers of interest or containing artifacts/errors after pre-processing. The high number of statistical tests requires corresponding correction (i.e., reduction of type I errors) by using the Holm-Bonferroni method. However, in line with previous recommendations [65,66] a trade-off between type I and type II errors (i.e., the probability of accepting the null hypothesis when the alternative is true) should be maintained. Therefore, we corrected only test series with more than 20 observations, meaning that for a threshold α = 5% one false positive would be expected.

## Supporting Information

S1 Data Excel spreadsheet containing, in separate sheets, the underlying numerical data for Figs 1B, 1C, 2B, 2F, 2G, 2H, 3A, 3B, 4C, 4F, 5B, 6C, 6F, 7, 8C, 8D, 8E and 8F. (XLSX)

S1 Fig Quantification of cross-modal modulation of induced network activity in the S1. (**A**) Baseline-normalized Morlet wavelet spectra of LFPs in the granular S1 layer after tactile (top) and bimodal stimulation (bottom) when averaged for all CON rats. Stimulus is marked by dotted gray line and arrows (tactile, green; bimodal, red). (**B**) Scatter plot of frequency-power coefficients calculated in (A) that significantly differed after bimodal stimulation when compared with unimodal (tactile) stimulation. Clusters (>50 points) served for defining time-windows (I, II and III) during which the modulation of induced network activity in gamma frequency band (dotted black boxes) differed between unimodal and bimodal conditions. (**C**) Baseline-normalized Morlet wavelet spectra displayed in (A) when superimposed with the time-windows I, II and III (dotted black boxes) identified in (B). (**D**) Bar diagrams displaying the relative power averaged during time-windows I, II and III for tactile (top) and bimodal stimulation (bottom). (**E**) Bar diagram displaying the difference between bimodal and tactile mean relative power during the time-windows I, II, and III. Significance values correspond to *p* < 0.05 (*) and *p* < 0.01 (**).
(TIF)

S2 Fig Quantification of cross-modal effects on MUA discharge over all layers of S1 in CON and NWT rats. (**A**) Bar diagrams displaying the absolute amplitude of MUA signal during a time window of 50 ms after a visual (yellow), tactile (green), and bimodal (red) stimulation when averaged for all CON rats (*n* = 10 rats). (**B**) Same as (A) for NWT rats (*n* = 9).
(TIF)

S3 Fig Quantification of visually induced phase reset of spontaneous oscillatory activity in S1. (**A**) LFP traces (4–12 Hz band-pass filtered) of 20 visual trials in the granular S1 layer of a P19 CON rat. Note the rather disorganized temporal structure of the spontaneous oscillations before stimulation (dotted gray line, black arrow) and their alignment after stimulus. (**B**) Examples of phase distribution histograms for the spontaneous oscillations during the 20 trials shown in (A) during randomly selected pre-stimulus (left) and post-stimulus (right) time windows marked in light blue in (A). The mean resultant vector is plotted in red. Note that the pre-stimulus phases are randomly distributed, while the post-stimulus phases showed a pronounced phase concentration. (**C**) Histogram of the length of resultant vectors at all time-points before and after stimulation (black arrow, dotted black line) displayed for the 20 visual trials shown in (A). The pre- and post-stimulus time-windows detailed in (B) are marked in light blue.
(TIF)

S4 Fig Illustration of staining quantification for all investigated CON and NWT rats. (**A**) Photographs depicting the V1 neurons in 100 μm-thick coronal slices that were retrogradely stained after FG injection into S1 of 7 CON rats. The red box marks the area with the highest density of stained neurons that was used for quantification. (**B**) Same as (A) for retrogradely stained neurons in NWT rats (*n* = 5).
(TIF)

S5 Fig Quantification of cross-modal influence on synchronized oscillatory activity between S1 and V1. (**A**) Histogram displaying the difference between post-stimulus (C_B_) and pre-stimulus coherence (C_PB_) between S1 and V1 (G layers) of CON rats after bimodal stimulation. (**B**) Same as (A) for tactile (top) and visual stimulation conditions (middle). To correct for the artificially high coherence resulting from the similar shapes of EPs, the coherence between shuffled tactile responses in S1 and visual responses in V1 was calculated (bottom). (**C**) Corrected cross-modal coherence-modification (C_S_) quantified as the difference between the spectrum displayed in (A) and the sum of relative coherence spectra displayed in (B). Dotted black lines mark the frequency range from 12–80 Hz for which the strongest modulation was found. (**D**) Mean relative coherence of the frequency range marked in (C). Significance value corresponds to *p* < 0.05 (*).
(TIF)

S6 Fig Pre-processing procedure for Granger causality analysis and validation of the model. (**A**) LFP traces of 100 bimodal trials in the granular S1 layer of a P19 CON rat (gray traces) and the ensemble mean (magenta) before preprocessing (i), after removing linear trends and temporal mean (ii) and after removing the ensemble mean (iii). (**B**) Akaike and Bayesian Information Criterion (AIC, blue; BIC, green) at different model orders for the data from (A). Chosen model order is marked by the red window. (**C**) Consistency of bivariate models for all pairs of supragranular, granular and infragranular layers of S1 and V1. The lower border for valid models is marked by the dashed red line. (**D**) Correlation of residual noise in bivariate models for all pairs of signals recorded in the supragranular, granular and infragranular layers of S1 and V1. The upper border for valid models is marked by the dashed red line. (**E**) Stability index for bivariate models for all pairs of signals recorded in the supragranular, granular, and infragranular layers of S1 and V1. The upper border for valid models is marked by the dashed red line.
(TIF)

S7 Fig Schematic diagrams of the temporal organization of behavioral experiments in continuous and discrete settings. (**A**) Left, schematic drawing of the manipulation/testing performed from P0–25 in CON and NWT rats used for continuous setting. The colored numbers correspond to the behavioral investigations marked in the box (right). (**B**) Same as (A) for CON and NWT rats tested in the discrete setting.
(TIF)

S1 Table Summary of statistics for all experiments. (**A**) Statistical testing, number of investigated rats and p-values for the analyses displayed in Fig 1. (**B**)–(**H**) Same as (A) for analyses in Figs 2–8.
(XLSX)

S2 Table Distribution of different objects over the conditions and settings of NOR in CON and NWT rats. (**A**) Photographs displaying the objects used for testing of novel object recognition. The numbers correspond to those mentioned in the tables. (**B**) Table summarizing the object distribution in the continuous setting. (**C**) Table summarizing the object distribution in the discrete setting.
(XLSX)

## Abbreviations

CON: control
COX: cytochrome oxidase
CSD: current source density
EP: evoked potential
FG: Fluorogold
G: granular
I: infragranular
LFP: local field potential
MUA: multi-unit activity
N1: first negative peak
NOR: novel object recognition
NWT: neonatal whisker-trimmed
P: postnatal day
P1: first positive peak
PFA: paraformaldehyde
S: supragranular
S1: primary somatosensory cortex
SC: superior colliculus
V1: primary visual cortex
