# Supplementary figures and images for: Neonatal Restriction of Tactile Inputs Leads to Long-Lasting Impairments of Cross-Modal Processing
Source: PLoS Biol. 2015 Nov 24;13(11):e1002304. doi: 10.1371/journal.pbio.1002304 (PMC4658190; doi:10.1371/journal.pbio.1002304)

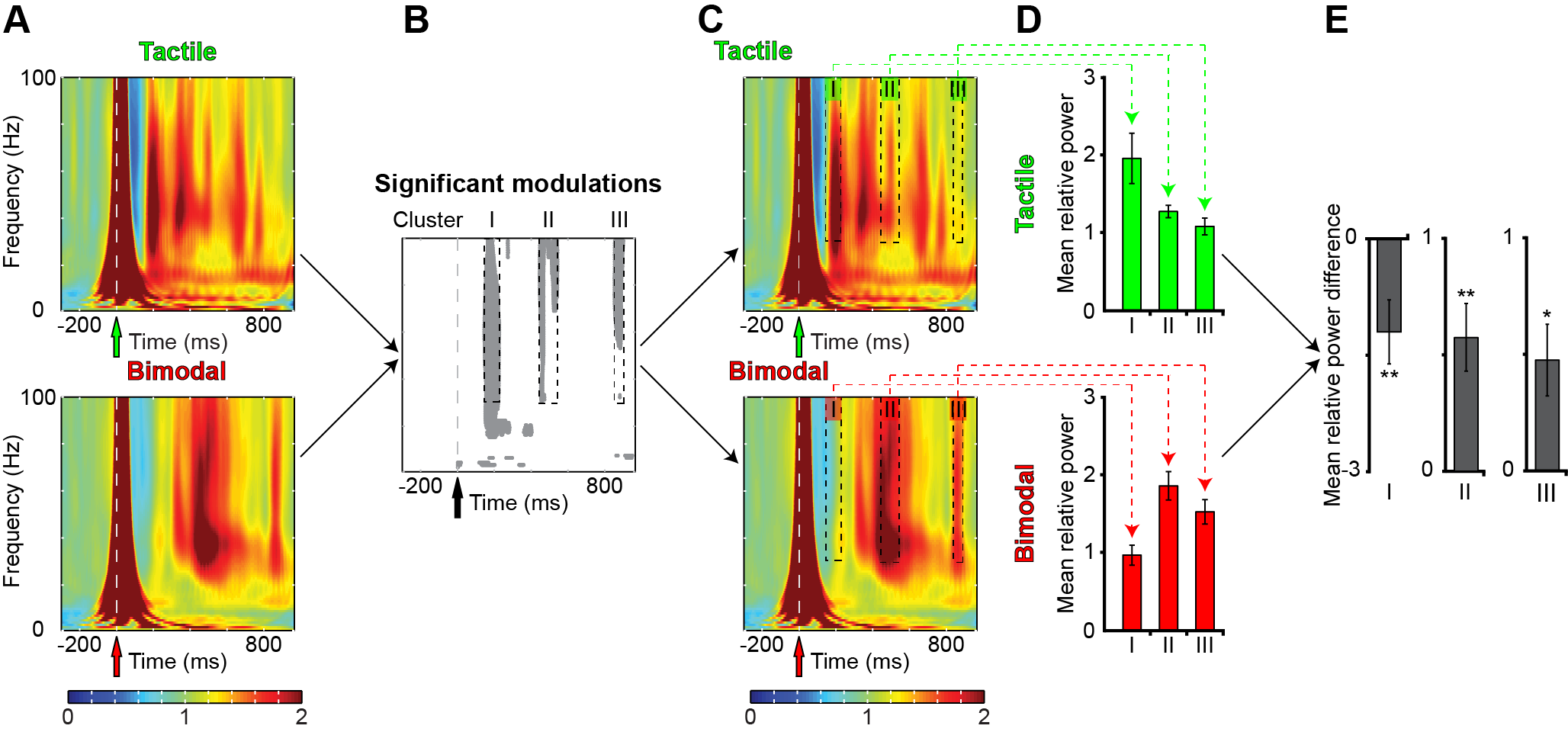

Supplement: S1 Fig — (A) Baseline-normalized Morlet wavelet spectra of LFPs in the granular S1 layer after tactile (top) and bimodal stimulation (bottom) when averaged for all CON rats. Stimulus is marked by dotted gray line and arrows (tactile, green; bimodal, red). (B) Scatter plot of frequency-power coefficients calculated in (A) that significantly differed after bimodal stimulation when compared with unimodal (tactile) stimulation. Clusters (>50 points) served for defining time-windows (I, II and III) during which the modulation of induced network activity in gamma frequency band (dotted black boxes) differed between unimodal and bimodal conditions. (C) Baseline-normalized Morlet wavelet spectra displayed in (A) when superimposed with the time-windows I, II and III (dotted black boxes) identified in (B). (D) Bar diagrams displaying the relative power averaged during time-windows I, II and III for tactile (top) and bimodal stimulation (bottom). (E) Bar diagram displaying the difference between bimodal and tactile mean relative power during the time-windows I, II, and III. Significance values correspond to p < 0.05 (*) and p < 0.01 (**). (TIF) [file pbio.1002304.s002.tif]

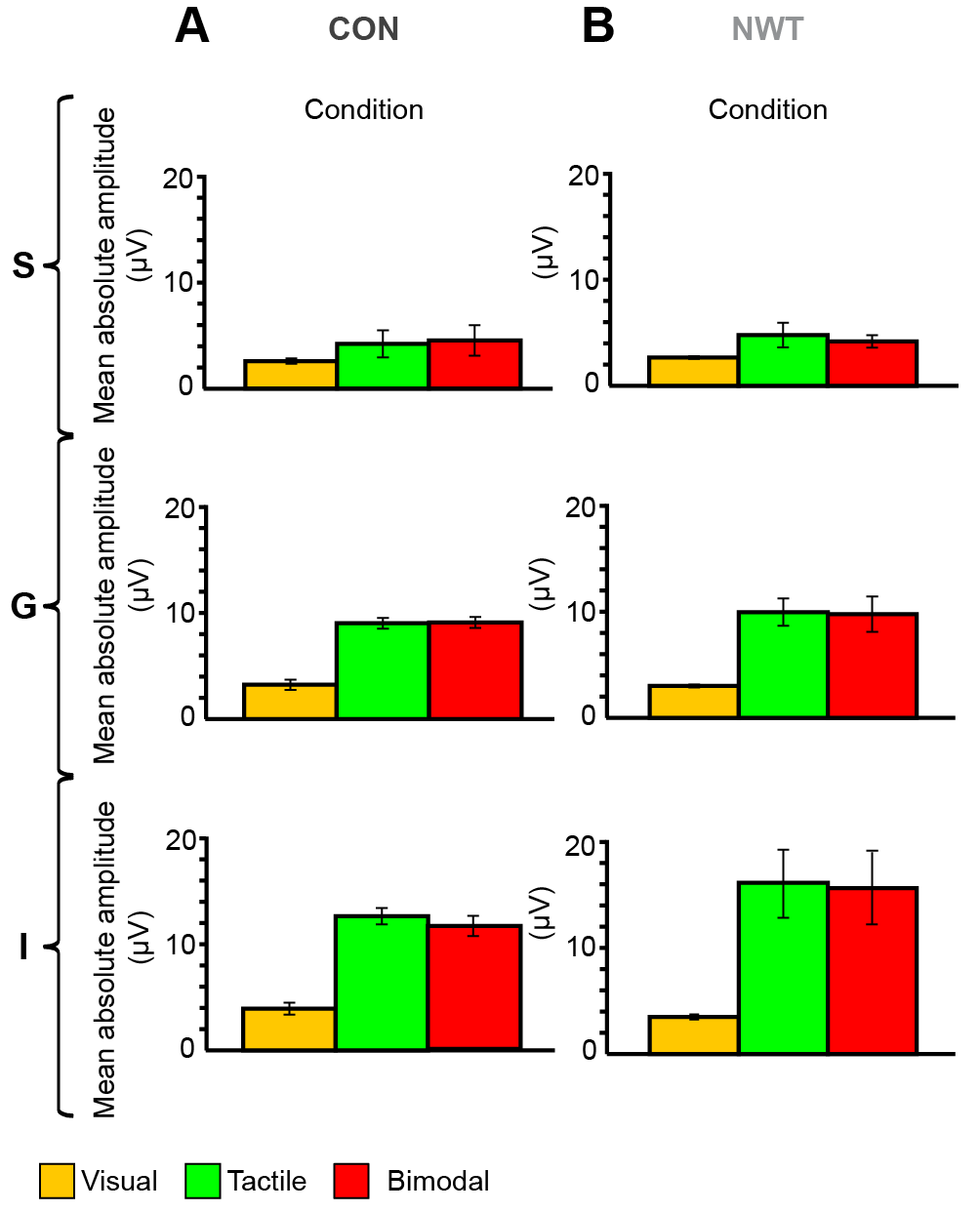

Supplement: S2 Fig — (A) Bar diagrams displaying the absolute amplitude of MUA signal during a time window of 50 ms after a visual (yellow), tactile (green), and bimodal (red) stimulation when averaged for all CON rats (n = 10 rats). (B) Same as (A) for NWT rats (n = 9). (TIF) [file pbio.1002304.s003.tif]

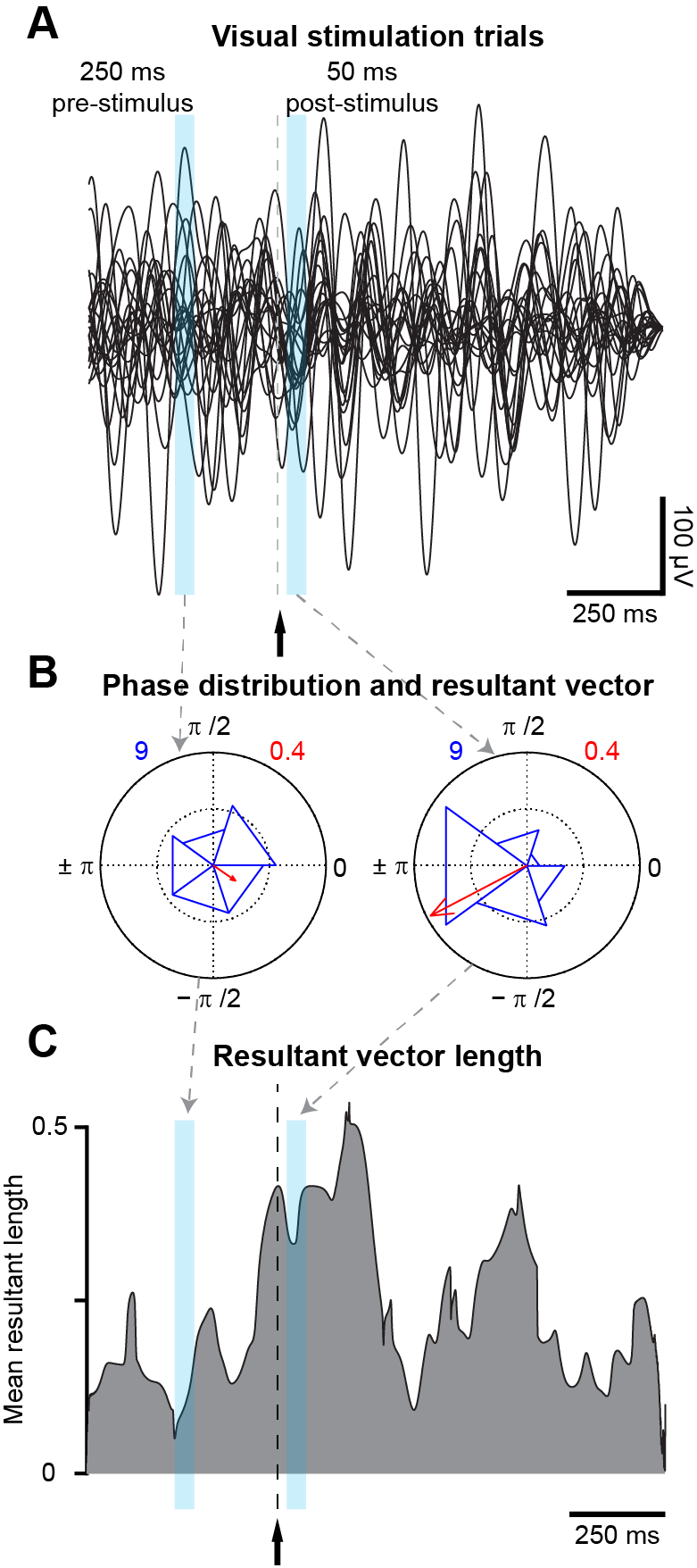

Supplement: S3 Fig — (A) LFP traces (4–12 Hz band-pass filtered) of 20 visual trials in the granular S1 layer of a P19 CON rat. Note the rather disorganized temporal structure of the spontaneous oscillations before stimulation (dotted gray line, black arrow) and their alignment after stimulus. (B) Examples of phase distribution histograms for the spontaneous oscillations during the 20 trials shown in (A) during randomly selected pre-stimulus (left) and post-stimulus (right) time windows marked in light blue in (A). The mean resultant vector is plotted in red. Note that the pre-stimulus phases are randomly distributed, while the post-stimulus phases showed a pronounced phase concentration. (C) Histogram of the length of resultant vectors at all time-points before and after stimulation (black arrow, dotted black line) displayed for the 20 visual trials shown in (A). The pre- and post-stimulus time-windows detailed in (B) are marked in light blue. (TIF) [file pbio.1002304.s004.tif]

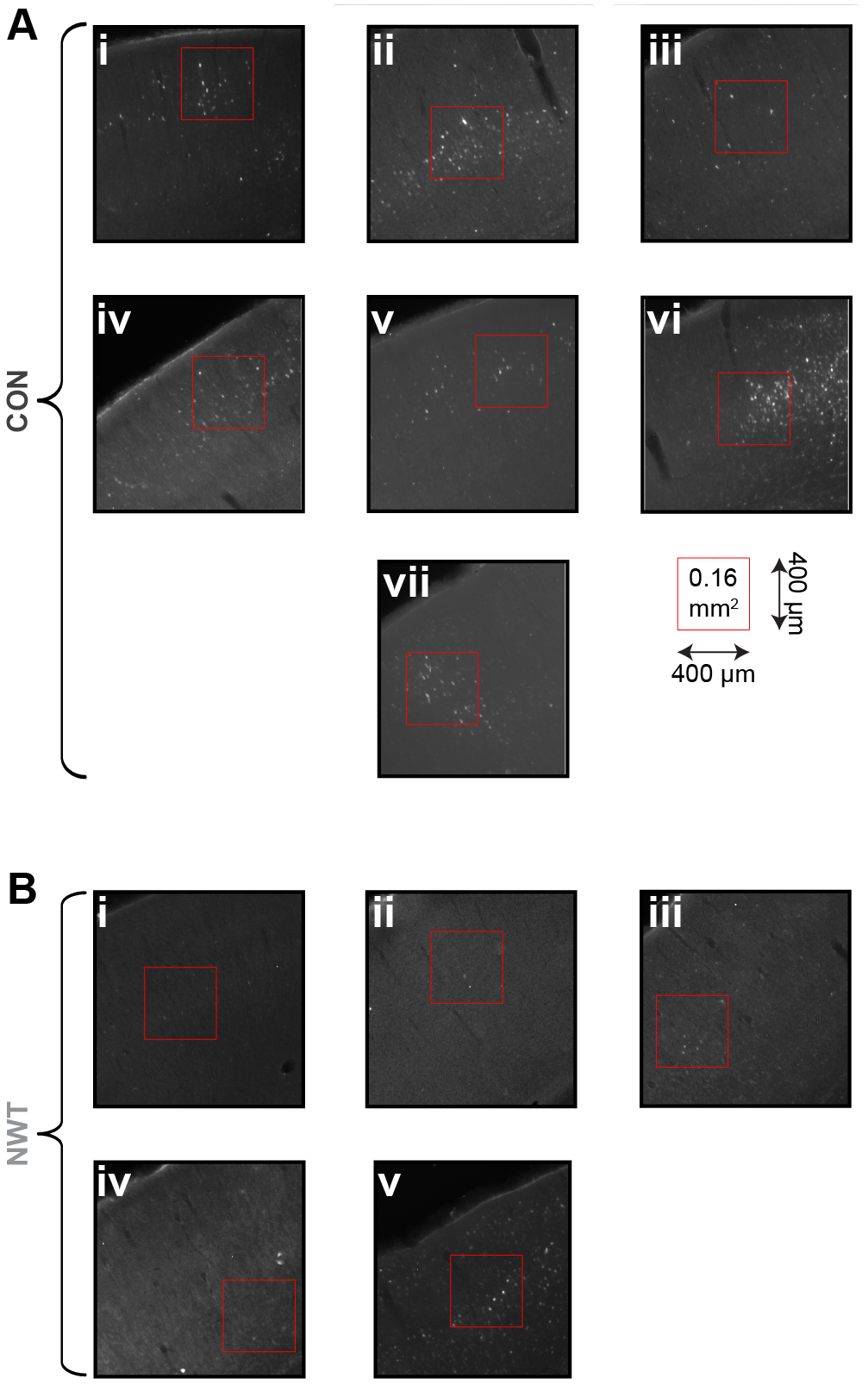

Supplement: S4 Fig — (A) Photographs depicting the V1 neurons in 100 μm-thick coronal slices that were retrogradely stained after FG injection into S1 of 7 CON rats. The red box marks the area with the highest density of stained neurons that was used for quantification. (B) Same as (A) for retrogradely stained neurons in NWT rats (n = 5). (TIF) [file pbio.1002304.s005.tif]

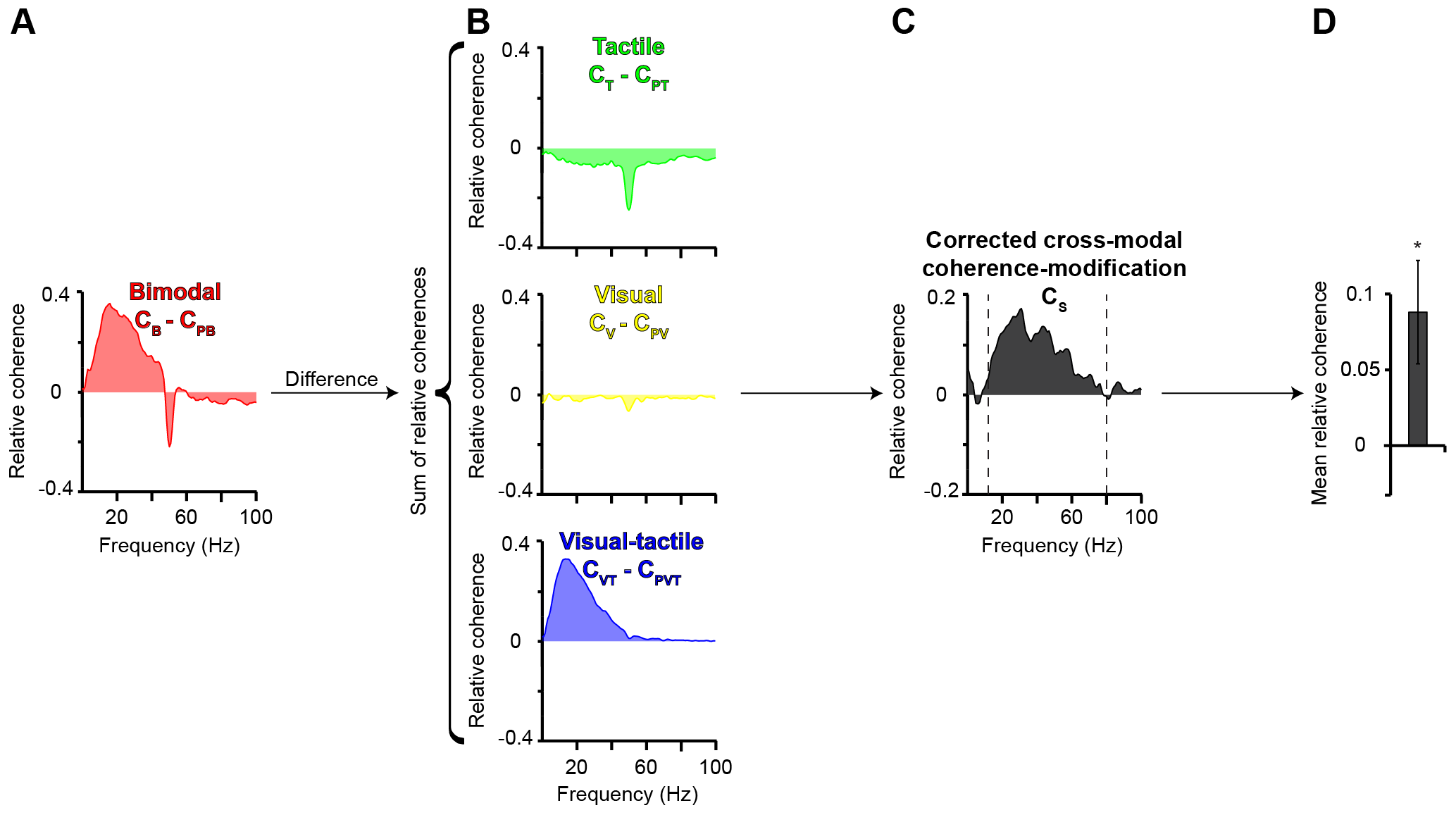

Supplement: S5 Fig — (A) Histogram displaying the difference between post-stimulus (CB) and pre-stimulus coherence (CPB) between S1 and V1 (G layers) of CON rats after bimodal stimulation. (B) Same as (A) for tactile (top) and visual stimulation conditions (middle). To correct for the artificially high coherence resulting from the similar shapes of EPs, the coherence between shuffled tactile responses in S1 and visual responses in V1 was calculated (bottom). (C) Corrected cross-modal coherence-modification (CS) quantified as the difference between the spectrum displayed in (A) and the sum of relative coherence spectra displayed in (B). Dotted black lines mark the frequency range from 12–80 Hz for which the strongest modulation was found. (D) Mean relative coherence of the frequency range marked in (C). Significance value corresponds to p < 0.05 (*). (TIF) [file pbio.1002304.s006.tif]

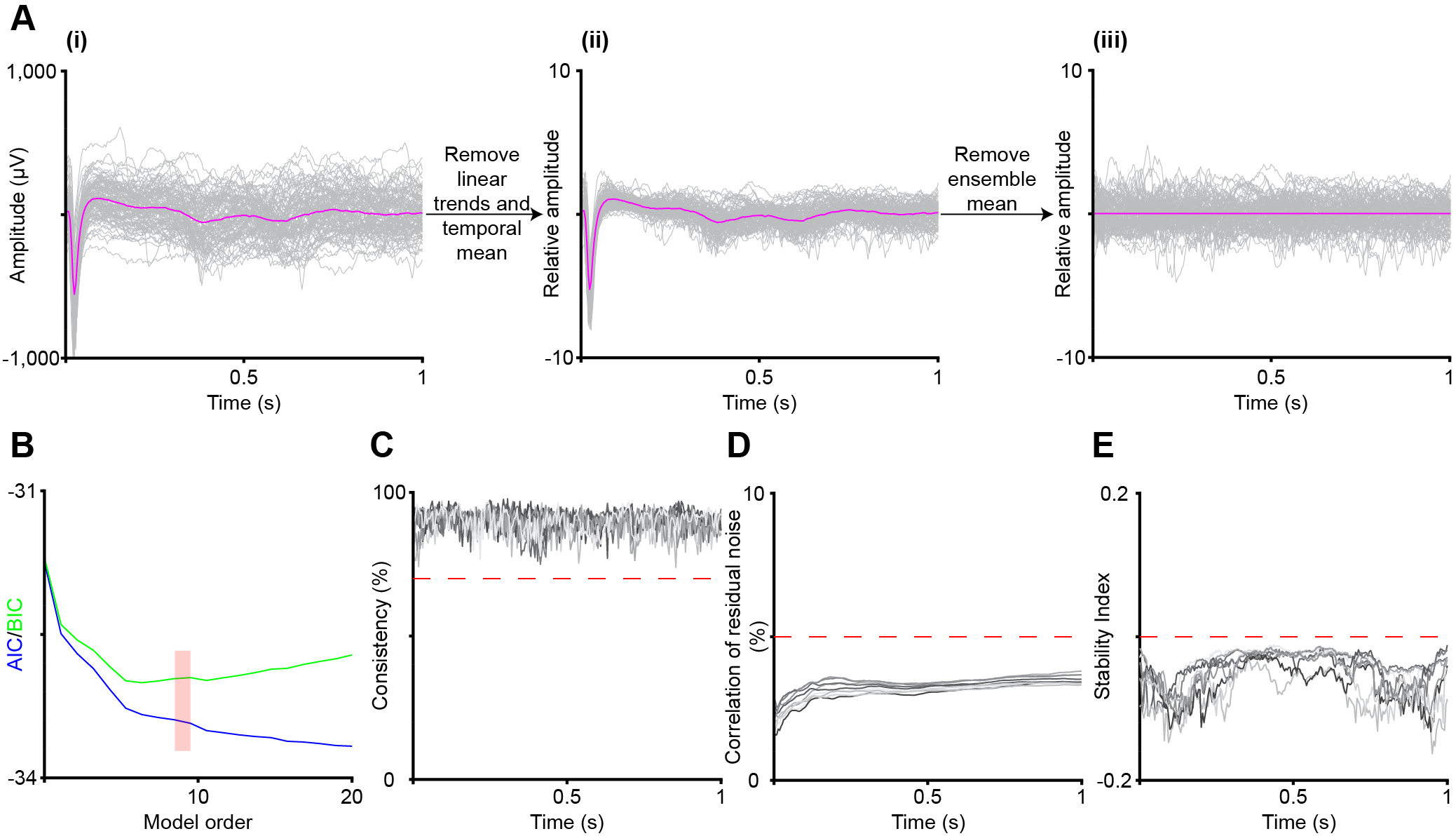

Supplement: S6 Fig — (A) LFP traces of 100 bimodal trials in the granular S1 layer of a P19 CON rat (gray traces) and the ensemble mean (magenta) before preprocessing (i), after removing linear trends and temporal mean (ii) and after removing the ensemble mean (iii). (B) Akaike and Bayesian Information Criterion (AIC, blue; BIC, green) at different model orders for the data from (A). Chosen model order is marked by the red window. (C) Consistency of bivariate models for all pairs of supragranular, granular and infragranular layers of S1 and V1. The lower border for valid models is marked by the dashed red line. (D) Correlation of residual noise in bivariate models for all pairs of signals recorded in the supragranular, granular and infragranular layers of S1 and V1. The upper border for valid models is marked by the dashed red line. (E) Stability index for bivariate models for all pairs of signals recorded in the supragranular, granular, and infragranular layers of S1 and V1. The upper border for valid models is marked by the dashed red line. (TIF) [file pbio.1002304.s007.tif]

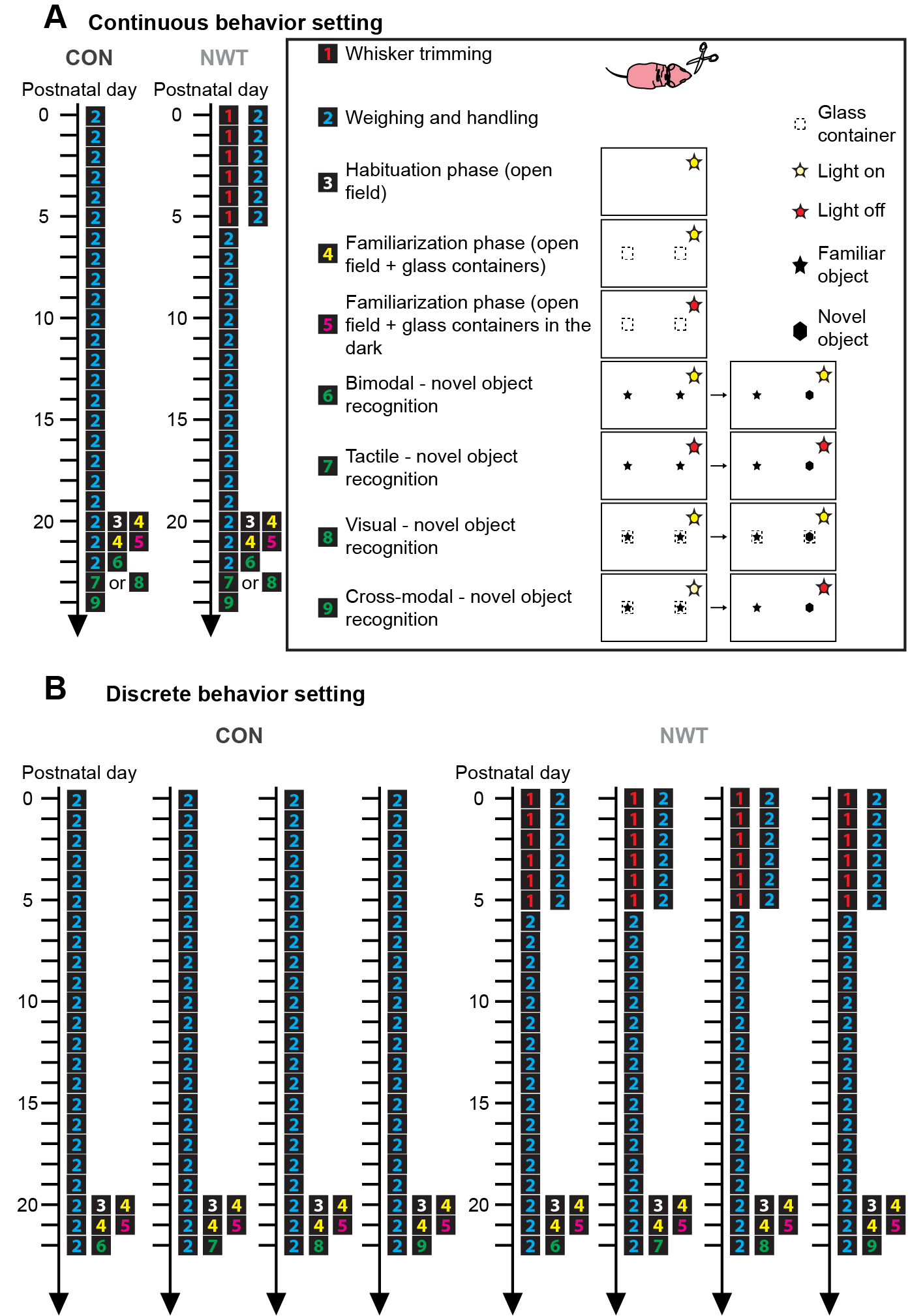

Supplement: S7 Fig — (A) Left, schematic drawing of the manipulation/testing performed from P0–25 in CON and NWT rats used for continuous setting. The colored numbers correspond to the behavioral investigations marked in the box (right). (B) Same as (A) for CON and NWT rats tested in the discrete setting. (TIF) [file pbio.1002304.s008.tif]
